# Switch-like phosphorylation of WRN integrates end-resection with RAD51 metabolism at collapsed replication forks

**DOI:** 10.1093/nar/gkae807

**Published:** 2024-09-24

**Authors:** Valentina Palermo, Eva Malacaria, Maurizio Semproni, Serena Camerini, Marialuisa Casella, Benedetta Perdichizzi, Pasquale Valenzisi, Massimo Sanchez, Federica Marini, Achille Pellicioli, Annapaola Franchitto, Pietro Pichierri

**Affiliations:** Department of Environment and Health, Mechanisms, Biomarkers and Models Section, Genome Stability Group, Istituto Superiore di Sanità, Viale Regina Elena 299, 00161 Rome, Italy; Department of Environment and Health, Mechanisms, Biomarkers and Models Section, Genome Stability Group, Istituto Superiore di Sanità, Viale Regina Elena 299, 00161 Rome, Italy; Department of Environment and Health, Mechanisms, Biomarkers and Models Section, Genome Stability Group, Istituto Superiore di Sanità, Viale Regina Elena 299, 00161 Rome, Italy; FAST, Core Facilities Service, Istituto Superiore di Sanità, Viale Regina Elena 299, 00161 Rome, Italy; FAST, Core Facilities Service, Istituto Superiore di Sanità, Viale Regina Elena 299, 00161 Rome, Italy; Department of Environment and Health, Mechanisms, Biomarkers and Models Section, Genome Stability Group, Istituto Superiore di Sanità, Viale Regina Elena 299, 00161 Rome, Italy; Department of Environment and Health, Mechanisms, Biomarkers and Models Section, Genome Stability Group, Istituto Superiore di Sanità, Viale Regina Elena 299, 00161 Rome, Italy; FAST, Core Facilities Service, Istituto Superiore di Sanità, Viale Regina Elena 299, 00161 Rome, Italy; Department of Biosciences, Genomic Instability and Human Pathologies Section, Università degli Studi di Milano, Via Giovanni Celoria 26, 20133 Milan, Italy; Department of Biosciences, Genomic Instability and Human Pathologies Section, Università degli Studi di Milano, Via Giovanni Celoria 26, 20133 Milan, Italy; Department of Environment and Health, Mechanisms, Biomarkers and Models Section, Genome Stability Group, Istituto Superiore di Sanità, Viale Regina Elena 299, 00161 Rome, Italy; Department of Environment and Health, Mechanisms, Biomarkers and Models Section, Genome Stability Group, Istituto Superiore di Sanità, Viale Regina Elena 299, 00161 Rome, Italy; Istituto Nazionale di Biostrutture e Biosistemi, Viale delle Medaglie d’Oro 305, 00134 Rome, Italy

## Abstract

Replication-dependent DNA double-strand breaks are harmful lesions preferentially repaired by homologous recombination (HR), a process that requires processing of DNA ends to allow RAD51-mediated strand invasion. End resection and subsequent repair are two intertwined processes, but the mechanism underlying their execution is still poorly appreciated. The WRN helicase is one of the crucial factors for end resection and is instrumental in selecting the proper repair pathway. Here, we reveal that ordered phosphorylation of WRN by the CDK1, ATM and ATR kinases defines a complex regulatory layer essential for correct long-range end resection, connecting it to repair by HR. We establish that long-range end resection requires an ATM-dependent phosphorylation of WRN at Ser1058 and that phosphorylation at Ser1141, together with dephosphorylation at the CDK1 site Ser1133, is needed for the proper metabolism of RAD51 foci and RAD51-dependent repair. Collectively, our findings suggest that regulation of WRN by multiple kinases functions as a molecular switch to allow timely execution of end resection and repair at replication-dependent DNA double-strand breaks.

## Graphical Abstract

**Graphical Abstract:**
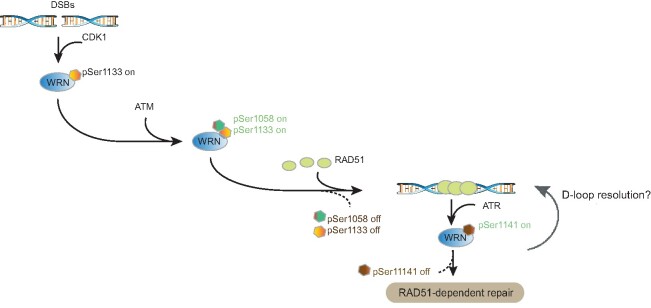

## Introduction

DNA double-strand breaks (DSBs) represent a major threat to genome integrity. They can be produced by physical and chemical agents, such as DNA topoisomerases poisons camptothecin and etoposide (1), or induced at stalled replication forks after their collapse by specialized endonucleases (2). In eukaryotes, DSBs formed during S-phase are repaired by homologous recombination (HR). However, these breaks must be processed to yield an intermediate suitable for RAD51-mediated strand invasion (3,4). The processing of DSBs involves various proteins necessary to carry out kilobase-long resection at the DNA ends (5–7). This extensive end resection, on one hand, inhibits the activation of proteins involved in the non-homologous end-joining (NHEJ) or microhomology-mediated end-joining (MMEJ) pathways of DSBs repair and, on the other hand, allows the loading of HR factors (3,8–10). Additionally, extensive long-range end resection triggers the activation of the Ataxia Telangiectasia-Related (ATR)-mediated signalling (11–13). Interestingly, while end-processing at DSBs is regulated by the Ataxia Telangiectasia-Mutated (ATM) protein and primarily the Cyclin-Dependent Kinase 1 (CDK1), repair by HR is regulated by ATR. This suggests that a switch between these two major regulatory circuits must occur (12,13). Although this molecular switch is expected to involve the assembly and disassembly of distinct molecular complexes, the exact mechanism and the proteins involved are only partially understood.

Among the proteins that work at the interface between these two phases of DSBs repair by HR, Werner syndrome protein (WRN) is particularly interesting. WRN is involved in several processes related to genome maintenance, and its loss leads to hypersensitivity to agents inducing DSBs, especially those acting during S-phase (14–18). WRN’s activities impact HR at multiple levels, including end resection and the pre-synaptic or post-synaptic metabolism of RAD51 (18,19). During long-range end resection, WRN is regulated by the CDK1 kinase, and its phosphorylation is necessary for proper HR repair (20). However, WRN is also targeted by ATM and ATR kinases (21,22) and partners with BRCA1 (23). Notably, the Breast Cancer-Associated protein 1 (BRCA1) is required for accurate end resection and repair and is also targeted by CDK1, ATM and ATR kinases (13,24). Hence, WRN is an ideal candidate for supporting the transition between the two phases of DSB repair by HR. Unfortunately, loss of WRN leads to complex phenotypes in response to DSBs, as its absence is partially offset by the Bloom syndrome protein (BLM) during end resection and HR (25–27).

In this study, we used WRN mutants to investigate the cross-talk between CDK1- and ATM/ATR-dependent WRN regulation in response to DSB formation and to assess whether WRN regulation coordinates end resection with the initiation of HR repair. Our results demonstrate that CDK1 and ATM regulation is an ordered process essential for establishing long-range end resection. We show that phosphorylation by CDK1 at Ser1133 of WRN stimulates modification at Ser1058 by ATM, which is crucial for correct resection by WRN. Notably, these ‘pro-resection’ sites must be turned off to promote ATR-dependent modification at Ser1141, which depends on long-range resection and allows proper metabolism of RAD51 foci and HR. Deregulated phosphorylation by ATR leads to inappropriate modification at Ser1133, preventing productive RAD51 foci formation and repair of DSBs.

Therefore, by using regulatory mutants of WRN that act as separation-of-function forms, we reveal a critical role of WRN as a molecular switch, facilitating the transition through different stages of HR at collapsed replication forks. Moreover, our findings shed light on how post-synaptic RAD51 metabolisms is regulated in human cells in response to replication-dependent, single-ended DSBs.

## Materials and methods

### Cell lines and culture conditions

The SV40-transformed WRN-deficient fibroblast cell line (AG11395-WS) was obtained from Coriell Cell Repositories (Camden, NJ, USA). To produce stable cell lines, AG11395 (WS) fibroblasts were transduced with lentiviruses expressing the full-length cDNA encoding wild-type WRN (WRN^WT^), S1133A-WRN (WRN^S1133A^), S1141A-WRN (WRN^S1141A^) and S1141D-WRN (WRN^S1141D^). HEK293T cells were obtained from American Type Culture Collection. HEK293TshWRN cells were generated by transfection with pRS-puro-shWRN (5′-AGGCAGGTGTAG- GAATTGAAGGAGATCAG-3′; sequence ID: TI333414 Origene) followed by puromycin selection (20). All cell lines were maintained in Dulbecco’s modified Eagle’s medium (DMEM; Life Technologies) supplemented with 10% FBS (Boehringer Mannheim) and incubated at 37°C in a humidified 5% CO_2_ atmosphere.

### Chemicals

Camptothecin (ENZO Life Sciences) was added to the culture medium at a concentration of 5 μM, if not otherwise specified, to induce single-ended DSBs. Mirin (Calbiochem), an inhibitor of MRE11 exonuclease activity, was used at a concentration of 50 μM. The B02 compound (Selleck), an inhibitor of RAD51 activity, was used at a final concentration of 27 μM. Roscovitine (Selleck), a pan-CDKs inhibitor, was used at a final concentration of 20 μM. ATM inhibitor (KU-55933, Selleck) and ATR inhibitor (VE-821, Selleck) were used at a final concentration of 10 μM. An inhibitor of DNA2 nuclease (28) was used at a final concentration of 300 μM. NU7441 (Selleck), a DNAPKcs inhibitor, was used at a final concentration of 1 μM, while the Ligase I/III L67 inhibitor (Sigma-Aldrich) was used at a final concentration of 6 μM. IdU (Sigma-Aldrich) was dissolved in sterile DMEM to make a 2.5 mM stock solution and stored at −20°C.

### Oligos and plasmids

The plasmids pCMV-FlagRNAi-resWRN^WT^, pCMV-FlagRNAi-resWRN^S1133A^ and pCMV-FlagRNAi-resWRN^S1133D^ were generated as described previously (20). Site-directed mutagenesis (SDM) was used to create the pCMV-FlagRNAi-resWRN mutant constructs. The Flag-ATM expression plasmid was described in reference (21), and the plasmid sgCAS9 was described in reference (29).

### Transfections

The pCMV-tag2B (pFlag) empty vector, pCMV-FlagRNAi-resWRN^WT^ or other pCMV-FlagRNAi-resWRN mutant constructs, as indicated, were transfected into HEK293TshWRN cells using DreamFect (OZ Bioscience) according to manufacturer’s instructions for 48 h.

The pCMV-tag2B (pFlag) empty vector, pCMV-FlagRNAi-resWRN^WT^, pCMV-FlagRNAi-resWRN^S1058A^, pCMV-FlagRNAi-resWRN^S1058D^, pCMV-FlagRNAi-resWRN^S1292A^, pCMV-FlagRNAi-resWRN^S1292D^ and pCMV-FlagRNAi-resWRN^2A^ constructs were transiently transfected into WRN-deficient fibroblast cell line using Neon transfection system 48 h prior to performing experiments.

The Cas9 plasmid without a guide RNA was transiently transfected either alone or in combination with pCMV-FlagRNAi-resWRN^WT^ or other pCMV-FlagRNAi-resWRN mutant constructs, as indicated. CtIP, RAD51 or BRCA1 siRNA was transfected at a final concentration of 50 nM using Lullaby (OZ Bioscience), 48 h before the experiments, following manufacturer’s instructions.

### Immunoprecipitation and western blotting analysis

Immunoprecipitation experiments were performed using 2.5 × 10^6^ cells. Cells were lysed in RIPA buffer (0.1% SDS, 0.5% Na-deoxycholate, 1% NP40, 150 mM NaCl, 1 mM EDTA, 50 mM Tris/Cl, pH 8) supplemented with phosphatase inhibitors, protease inhibitors and benzonase. One milligram of lysate was incubated overnight at 4°C with 20 μl of Anti-Flag M2 magnetic beads (Sigma-Aldrich) or 20 μl of Dynabeads Protein G (Invitrogen) conjugated with anti-WRN antibody (Abcam). After extensive washing in RIPA buffer, proteins were released in 2X electrophoresis buffer and subjected to sodium dodecyl sulfate-polyacrylamide gel electrophoresis (SDS-PAGE) followed by western blotting.

Western blotting was performed using standard methods. The blots were incubated with the following primary antibodies: rabbit anti-WRN (Abcam, 1:2000), mouse anti-β-Tubulin (Sigma-Aldrich, 1:2000), rabbit anti-Lamin B1 (Abcam, 1:40 000), mouse anti-GAPDH (Millipore, 1:5000), mouse anti-DDK-Flag tag (Origene, 1:2000), rabbit anti-pS/TQ (Cell Signaling Technology, 1:1000), rabbit anti-pS1141WRN (Sigma-Aldrich, 1:1000), rabbit anti-pS1133WRN (Genscript-custom, 1:10 000; (20), rabbit anti-GST (Calbiochem, 1:5000) and anti-BRCA1 (Santa Cruz, 1:500). After incubation with horseradish peroxidase-linked secondary antibodies (1:30 000, Jackson Immunoscience), the blots were detected using the Western Bright ECL detection kit (Advansta) according to the manufacturer’s instructions. Quantification was performed on scanned images of the blots using Image Lab software, with values shown on the graphs normalized to protein content as evaluated through Lamin B1 or β-tubulin-immunoblotting.

### Mass spectrometry analysis

Immunoprecipitated samples, untreated or treated with CPT for 1 h and for 4 h followed by recovery, were purified on a gradient SDS-PAGE gel. The bands corresponding to Flag-WRN were cut and *in-gel* digested with trypsin at 37°C overnight. After cysteine reduction with dithiothreitol and alkylation with iodoacetamide, the peptide mixture was injected into an Ultimate 3000 UHPLC (Dionex, Thermo Fisher Scientific) coupled with an Orbitrap Fusion Tribrid mass spectrometer (Thermo Fisher Scientific, CA, USA). Peptides were desalted on a trap column (Acclaim PepMap 100 C18, Thermo Fisher Scientific) and then separated on a 35 cm long silica capillary (Silica Tips, MSWil, The Netherlands), packed in-house with a C18, 1.9 μm, 100 Å resin (Michrom BioResources, CA, USA). The analytical separation was run for a 60 min gradient of buffer A (5% of acetonitrile and 0.1% of formic acid) and buffer B (95% of acetonitrile and 0.1% of formic acid). Buffer B was increased from 5% to 30% in 25 min, then to 80% in further 5 min, and 5 min of washing step (80% B) and 10 min of equilibrating step (5% B) were added to complete the run. Mass spectrometry (MS) analysis was initially performed in data-dependent acquisition (DDA), using 120 K and 60 K resolution in the Orbitrap for MS and MS2 acquisition, respectively. Raw data were analysed using Proteome Discoverer v 2.4 (Thermo). The database included the Flag-WRN sequence, with searches for specific tryptic peptides potentially oxidized on methionine and/or phosphorylated on serine, threonine or tyrosine. A tolerance of 10 ppm for precursor ions and 0.02 Da for fragment ions was applied, with a maximum Delta *C*n of 0.05. MS/MS spectra were manually verified. Subsequently, a parallel reaction monitoring (PRM) experiment was conducted. Precursor ions were selected based on the DDA results, and the transitions were analysed using Thermo Excalibur Qual Browser software (Table 1).

**Table 1.** List of settings used for analysis by the Thermo Excalibur Qual browser

WRN peptide	State	Charge	Precursor (m/z)	Product (*m/z*)
1128–1136	Unmodified	*z* = 2	509.77	818.41
	Phosphorylated	*z* = 2	549.75	800.40
1137–1159	Unmodified	*z* = 2	1263.64	1593.81
		*z* = 3	842.76	367.19
	Phosphorylated	*z* = 2	1304.12	1593.81
		*z* = 3	869.75	367.19

Analyses were performed on triplicate biological samples. The row MS data have been deposited in the ProteomeXchange Consortium via the MassIVE partner repository with the dataset identifier MSV000094766.

### Immunofluorescence assay

Cells were grown on 22 × 22 coverslips or 8-well Nunc chamber slides. To detect RAD51 foci, we performed pre-extraction on ice for 5 min in 0.5% Triton X-100 and then fixed with 3% paraformaldehyde (PFA)/2% sucrose at room temperature (RT) for 10 min. After blocking in 3% bovine serum albumin (BSA) for 15 min, staining was performed with rabbit polyclonal anti-RAD51 (Abcam, 1:1000) diluted in 1% BSA/0.1% saponin in PBS solution for 1 h at 37°C in a humidifier chamber. After extensive washing with PBS, a species-specific fluorescein-conjugated secondary antibody (Alexa Fluor 488-conjugated Goat Anti-Mouse IgG (H + L), highly cross-adsorbed; Life Technologies) was applied for 1 h at room temperature. Counterstaining was performed with 0.5 μg/ml 4,6-diamidino-2-phenylindole (DAPI). Secondary antibodies were used at 1:200 dilution. Slides were analysed with an Ecliplse 80i Nikon Fluorescence Microscope, equipped with a Video Confocal (ViCo) system at 60× magnification.

### Detection of nascent single-stranded DNA by native IdU assay

To detect nascent single-stranded DNA (ssDNA), cells were grown on 22 × 22 coverslips in 35 mm dishes. After 24 h, the cells were labeled for 15 min with 50 μM IdU (Sigma-Aldrich) before the treatment with 5 μM CPT for various time points.

For immunofluorescence cells were washed with PBS, permeabilized with 0.5% Triton X-100 for 10 min at 4°C and fixed with 2% sucrose, 3% PFA. Fixed cells were then incubated with primary mouse anti-IdU antibody (Becton Dickinson) for 1 h at 37°C in 1%BSA/PBS, followed by incubation with a species-specific fluorescein-conjugated secondary antibody (Alexa Fluor 488-conjugated Goat Anti-Mouse IgG (H + L), highly cross-adsorbed; Life Technologies). Nuclei were counterstained with 0.5 μg/ml 4,6-diamidino-2-phenylindole (DAPI).

Slides were analysed with an Eclipse 80i Nikon Fluorescence Microscope, equipped with a Virtual Confocal (ViCo) system. For each time point, at least 100 nuclei were scored at 40× magnification. Parallel samples incubated with the appropriate normal serum or only with the secondary antibody confirmed that the observed fluorescence pattern was not due to artifacts. Quantification was performed using ImageJ software.

### Generation of the GST-WRN fragment

The DNA sequence corresponding to amino acids 940–1432 (C-WRN) of WRN was amplified by PCR from either the pCMV-FlagWRN plasmid or the pCMV-FlagWRN^S1133D^ mutant. The PCR products were subsequently purified and subcloned into the pGEX4T-1 vector (Stratagene) for expression in bacteria as GST-fusion proteins. The resulting vectors were sequenced to ensure no mutations were introduced into the WRN sequence and were used to transform BL21 cells (Stratagene). Expression of GST and GST-fusion proteins was induced by the addition of 1 mM isopropyl-D-thiogalactopyranoside (IPTG) for 2 h at 37°C. GST and GST-C-WRN were affinity-purified using glutathione (GSH)-magnetic beads (Promega).

### *In vitro* kinase assay

For the kinase assay, 2 μg of immunopurified GST-tagged WRN fragment was phosphorylated *in vitro* by immunopurified Flag-ATM in the presence or absence of 5 μM ATP for 30 min at 37°C. After incubation, WRN fragments were separated from the beads. Phosphorylation levels were assessed by SDS-PAGE followed by Coomassie staining and densitometric analysis using phosphorimaging or western blotting with rabbit anti-pS/TQ antibody.

### Neutral comet assay

DNA breakage induction was evaluated using Comet assay (single cell gel electrophoresis) under non-denaturing condition. Briefly, dust-free frosted-end microscope slides were soaked in methanol overnight to remove fatty residues. The slides were then dipped into molten low melting point (LMP) agarose at 0.5% and allowed to dry. Cell pellets were resuspended in PBS and kept on ice to inhibit DNA repair. Cell suspensions were rapidly mixed with LMP agarose at 37°C, and an aliquot was spread onto the agarose-covered surface of the slide. The agarose-embedded cells were lysed by submerging the slides in lysis solution (30 mM ethylenediaminetetraacetic acid [EDTA], 0.1% SDS) and incubated at 4°C, 1 h in the dark. After lysis, slides were washed in 1X Tris Borate EDTA (TBE) running buffer (90 mM Tris, 90 mM boric acid, 4 mM EDTA) for 1 min. Electrophoresis was performed for 20 min in 1X TBE buffer at 1 V/cm. Slides were subsequently washed in distilled H_2_O and dehydrated in ice-cold methanol. Nuclei were stained with GelRed (Biotium, 1:1000) and visualized with a fluorescence microscope (Zeiss), using a 20× objective, connected to a CDD camera for image acquisition. At least 200 comets per cell line were analysed using Comet Assay IV software (Perceptive instruments). To assess the extent of DNA DSB breaks, computer generated tail moment values (tail length × fraction of total DNA in the tail) were used. Data from tail moments were processed using Prism software. Apoptotic cells (characterized by small comet head and extremely larger comet tail) were excluded from the analysis to avoid artificial enhancement of the tail moment.

### *In situ* PLA assay for ssDNA–protein interaction

*In situ* PLA (Merck) was performed according to the manufacturer’s instruction. For detecting nascent ssDNA–protein interactions, cells were labelled with 100 μM IdU for 15 min before treatments. Following treatment, cells were permeabilized with 0.5% Triton X-100 for 10 min at 4°C, fixed with 3% formaldehyde/2% sucrose solution for 10 min, and then blocked in 3% BSA/PBS for 15 min. After washing with PBS, cells were incubated with the relevant primary antibodies. The primary antibodies used were: rabbit monoclonal anti-RAD51 (Abcam, 1:1000) and mouse monoclonal anti-BrdU/IdU (Becton Dickinson, clone b44, 1:10). For negative control, only one primary antibody was used. Samples were incubated with secondary antibodies conjugated with PLA probes MINUS and PLUS: anti-mouse PLUS and anti-rabbit MINUS (Merck). Incubation with all antibodies was performed in a humidified chamber for 1 h at 37°C. The PLA probes MINUS and PLUS were ligated using connecting oligonucleotides to produce a template for rolling cycle amplification. Following amplification, the products were hybridized with a red fluorescence-labelled oligonucleotide. Samples were mounted in Prolong Gold anti-fade reagent with DAPI (blue). Images were acquired randomly using an Eclipse 80i Nikon Fluorescence Microscope, equipped with a Video Confocal (ViCo) system.

### Homologous recombination reporter assay

HEK293TshWRN cells (20) were seeded in six-well plates at a density of 0.5 million cells per well. The following day, cells were co-transfected with pCMV-FlagRNAi-resWRN^WT^ or other pCMV-FlagRNAi-resWRN mutant constructs, along with the I-SceI expression vector pCBASceI and the pHPRT-DRGFP plasmid reporter, using DreamFect (OZ Bioscience) according to the manufacturer’s instructions. The pHPRT-DRGFP and pCBASceI plasmids were gifts from Maria Jasin (Addgene plasmids # 26 476 and #26 477).

Protein expression levels were analysed by western blotting 72 h post-transfection. Additionally, flow cytometry analysis was performed 72 h after transfection to determine the percentage of GFP-positive cells.

### Human genomic DNA extraction

The SV40-transformed WRN-deficient fibroblast cell line was grown in six-well plates after transfection. At the indicated time points, cells were trypsinized, washed in PBS, and genomic DNA was extracted using the NucleoSpin™ Tissue kit (Macherey-Nagel), according to the manufacturer’s instructions. The following day, 15 μl of genomic DNA (approximately 100 ng/μl) were either digested with 20 units of *Bsr*GI or *Bam*HI restriction enzymes (New England BioLabs) or left undigested as a mock control, and incubated for 5 h at 37°C. The digested and mock DNA was purified, and 5 μl of each sample were used for the droplet digital PCR (ddPCR) reaction.

### Droplet digital PCR assay

The ddPCR reaction was prepared as follows: 5 μl of genomic DNA (approximately 50 ng), 1X ddPCR™ Supermix for Probes (no dUTP, Bio-Rad), 900 nM of each primers, 250 nM of each probe (Hexachloro-fluorescein, HEX and Fluorescein amidites, FAM) and dH_2_O to a total volume of 20 μl per sample. Droplets were generated by pipetting 20 μl of the PCR reaction mix into a single well of a universal DG8™ cartridge for droplets generation (Bio-Rad). Additionally, 70 μl of droplet generation oil was added to each well alongside the samples. Cartridges were covered with DG8™ droplet generator gaskets (Bio-Rad) and placed into the droplet generator (QX200™, Bio-Rad). After droplet generation, 40 μl of the emulsion were transferred from the right well of the cartridge to a 96-well ddPCR plate (BioRad). The 96-well PCR plate was sealed with peelable foil heat seals using the PCR plate sealer machine (PX1™, Bio-Rad).

PCR was performed using a T100™ Thermocycler (Bio-Rad) with a ramp rate of 2.5°C/s between each step. The protocol includes an initial activation at 95°C for 5 min, followed by 39 cycles of 95°C for 30 s and 58.7°C for 1 min. After cycling, the reaction was held at 4°C for 5 min and then at 90°C for 5 min, before being held at 12°C. Post-PCR luorescence was read using the QX200™ Droplet Reader (Bio-Rad) with QuantaSoft™ Software (Bio-Rad). On average, each sample generated approximately 15 000 droplets. The number of copies/μl of each target locus was determined by setting an empirical baseline threshold identical across the samples.

Cas9 cleavage efficiency and measurement of ssDNA were calculated as described in reference (29).

### Statistical analysis

Data are presented as the mean of at least two independent experiments. Statistical comparisons of WRN-deficient (WS) or WRN-mutant cells to their relevant controls were conducted by one-sided analysis of variance (ANOVA) for Comet assays, and the Mann–Whitney test for ssDNA, PLA and other experiments, employing the built-in tools in Prism 9 (GraphPad Inc.).

Statistical significance is denoted as follows: ns = not significant; *P < 0.05, **P < 0.01, ***P < 0.001, ****P < 0.0001. Detailed statistical analyses are provided in the respective figure legends.

## Results

### CDK1-dependent phosphorylation of WRN permits subsequent modification by ATM upon replication fork collapse

**Figure 1.**
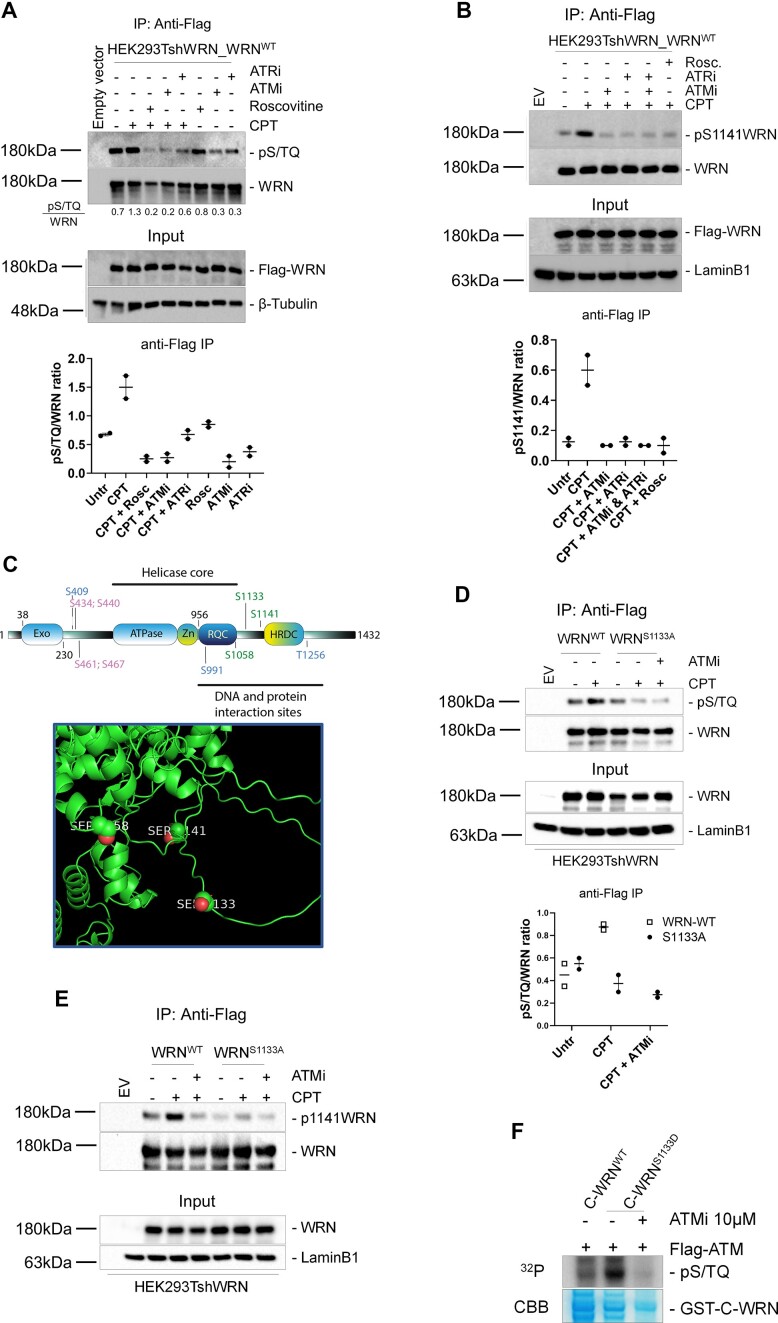
ATM/ATR-dependent WRN phosphorylation requires CDK activity upon CPT. (**A**) WRN was immunoprecipitated from cells transiently transfected with Flag-WRN wild-type (WRN^WT^) and treated with CDKi (Roscovitine), ATMi (KU-55933), ATRi (VE-821), alone or in combination, and then with CPT for 4 h. Nine-tenths of the IPs were analysed by western blotting (WB) with the anti-pS/TQ antibody, while 1/10 was analysed by anti-WRN antibody. Input represents 1/50 of the lysate. Anti-Flag antibody was used to verify transfection and an anti-β-tubulin antibody was used as a loading control. Quantification of the representative blots is reported below each lane. The graph represents quantification of the densitometry analysis from biological duplicates. (**B**) Cells were treated and subjected to anti-Flag-WRN IPs as in (A). Nine-tenths of IPs were subjected to WB with an anti-pS1141WRN antibody, while 1/10 was detected by anti-Flag antibody. Input represents 1/50 of the lysate. Anti-LaminB1 was used as a loading control. The graph represents quantification of the densitometry analysis from biological duplicates. (**C**) Schematic representation of the WRN protein with domains and localization of the three phosphorylation sites implicated in this study (S1058, S1133, S1141), the ATR-targeted sites (S409, S991, S1256) and the CK2-targeted sites (S434, 440, 461, 467) implicated in the response to replication arrest. Below is an AF3-modelling of WRN structure with the position of S1058, S1133 and S1141 in predicted unstructured structures. (**D**) Cells transiently transfected with WRN^WT^ or WRN^S1133A^ mutant were treated with 10 μM ATMi then with CPT for 4 h followed by IP/WB. Nine-tenths of IPs were analysed by WB with the anti-pS/TQ antibody, while 1/10 was analysed by anti-WRN antibody. Input represents 1/50 of the lysate. Anti-Flag was used to verify transfection and an anti-LaminB1 antibody was used as a loading control. The graph represents quantification of the densitometry analysis from biological duplicates. (**E**) Cells were treated and subjected to anti-Flag-WRN IPs as in (C). Nine-tenths of IPs were analysed by WB with the anti-pS1141WRN antibody. (**F**) *In vitro* ATM kinase assay. For kinase assay, 2 μg of immunopurified GST-tagged WRN wild-type fragment (C-WRN^WT^) or WRN phosphomimetic mutant fragment (C-WRN^S1133D^) were phosphorylated *in vitro* using Flag-tagged ATM kinase, immunoprecipitated with specific anti-Flag-conjugated beads. Immunoblotting was used to analyse the ATM-dependent phosphorylation level in different WRN fragments using an anti-pS/TQ antibody. Treatment with 10 μM ATM inhibitor (KU-55933) was used as a control. Coomassie (CBB) showed GST-C-WRN fragments in the gel as a control.

To test the possibility that WRN is phosphorylated at ATM consensus site(s) during DSBs processing, we expressed Flag-tagged wild-type WRN in HEK293TshWRN cells and treated them as reported (Figure 1A). Cell lysates were subjected to immunoprecipitation using an anti-Flag antibody, and the immunoprecipitates were examined by western blotting with an anti-pS/T-Q antibody, which recognizes ATM/ATR substrates. Under unperturbed conditions, both ATR and ATM contributed similarly to WRN modifications at S/TQ sites, while CDK did not (Figure 1A). Following CPT treatment, we observed increased WRN phosphorylation, which was more efficiently reduced by the ATM inhibitor (KU-55933) than by the ATR inhibitor (VE-821) (Figure 1A), suggesting that WRN phosphorylation is primarily ATM-dependent. Notably, phosphorylation at S/TQ motifs of WRN was also substantially reduced by the CDK inhibitor (Roscovitine) upon treatment (Figure 1A).

WRN contains six S/TQ sites, two of which, Ser1058 and Ser1141, are critical for ATM-dependent regulation (21,22). We focused our analysis on Ser1141, for which a commercially available anti-pS1141-WRN antibody exists. As shown in Figure 1B, CPT treatment strongly enhanced S1141-WRN phosphorylation. Notably, Ser1141 phosphorylation of WRN was similarly reduced by both ATM and ATR inhibitors and was markedly decreased after treatment with Roscovitine (Figure 1B). These results suggest that CDK-dependent phosphorylation is essential for enabling ATM/ATR-dependent regulation of WRN in response to CPT. Under our conditions, the DNA damage response inhibitors used did not induce any noticeable alteration in the cell cycle profile of the cells used for the IP/WB assays (Supplementary Figure S1). However, we acknowledge that the exclusive use of pharmacological inhibition of ATM and ATR may represent a limitation of this study, although it probably represents the safer way to avoid long-term inhibition and cell cycle effects.

Since ATM/ATR-dependent phosphorylation of WRN was not detectable after Roscovitine treatment, we examined the phosphorylation status of the regulatory CDK site Ser1133, which is essential for the role of WRN during end-resection (20) and is close to Ser1058 and Ser1141, to assess if a dependency existed. To test our hypothesis, we expressed the CDK1-unphosphorylable S1133A-WRN mutant (20) in HEK293TshWRN cells and treated them with or without CPT (Figure 1D). S/TQ phosphorylation was largely suppressed in the S1133A-WRN mutant in response to CPT but not under unperturbed conditions, compared with cells expressing wild-type WRN. Interestingly, a strong reduction in Ser1141 phosphorylation was observed in S1133A-WRN mutant cells after CPT treatment or ATM inhibition (Figure 1E). In agreement with the crucial role of Ser1133 phosphorylation of WRN in long-range resection (20), depletion of CtIP greatly reduced phosphorylation at Ser1141 (Supplementary Figure S2). This suggests that the effect of ATM inhibition on Ser1141 phosphorylation might be either direct or indirect, correlating with impairment of end resection.

Since most phosphorylation events on WRN S/TQ sites depend on ATM during CPT treatment, we next performed an *in vitro* ATM kinase assay using a C-terminal fragment of WRN as a substrate, either wild-type or containing the S1133D phosphomimetic mutation (Figure 1F). We found that the S1133D phosphomimetic mutation greatly increased phosphorylation by ATM. These findings indicate that CDK1 primes ATM-dependent modification of WRN at its target sites.

Notably, CDK1-dependent phosphorylation of WRN at S1133 was found to be dependent on ATM activity (Supplementary Figure S3A). Both ATM/ATR- and CDK1-dependent phosphorylation of WRN were also found to be both MRE11 nuclease-dependent (Supplementary Figure S3B), supporting that all modifications at these sites depend on end resection, a pathway in which ATM plays important regulatory roles.

Altogether, our results indicate that the response to single-ended DSBs (seDSBs), as induced by CPT treatment, requires an ordered phosphorylation of WRN by CDK1, ATM and/or ATR. They also demonstrated that all phosphorylation events depend on end resection, as they require CtIP and MRE11.

### Regulated and site-specific phosphorylation of WRN is crucial for end resection at DSBs

**Figure 2.**
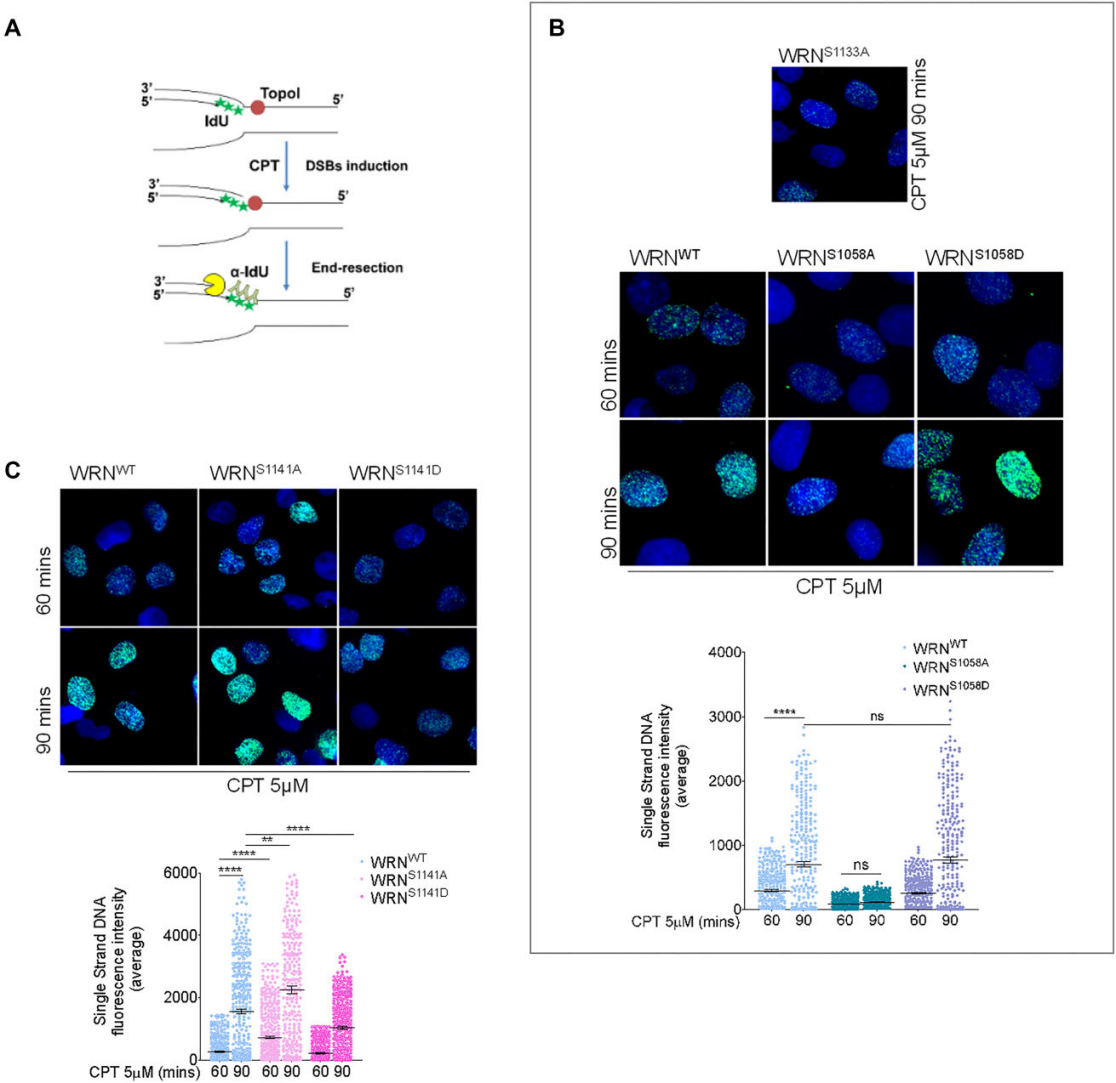
Phosphorylation by ATM/ATR of WRN at distinct sites differently affects end resection of DSBs. (**A**) Cartoon depicting the ssDNA assay by native anti-IdU immunofluorescence. The scheme shows how ssDNA can be visualized at collapsed replication forks. CPT treatment results in one-ended DSBs at replication forks, leading to 5′-3′ resection of template DNA by nucleases (pacman) thus exposing nascent ssDNA, which is detected by native IdU/ssDNA assay. Nascent DNA was pre-labelled for 15 min with IdU before treatment, and labelling remained during treatment with CPT. (**B** and **C**) WS fibroblasts were transiently transfected with the indicated WRN-expressing plasmid. The ssDNA was analysed at different time points, as indicated. Dot plots show the mean intensity of IdU/ssDNA staining for single nuclei (*n* = 300, two biological replicates). Data are presented as mean ± SE. Representative images of IdU/ssDNA-stained CPT-treated cells are shown. A panel showing ssDNA detected in the cells complemented with the end resection-defective S1133A-WRN mutant is shown as a reference. Statistical analysis was performed by the ANOVA test (****P< 0.0001, **P< 0.01, *P> 0.05; ns = not significant).

To determine whether ATM-dependent phosphorylation of WRN could be involved in the resection of DNA ends, we evaluated the formation of ssDNA using the IdU/ssDNA assay (Figure 2A). End resection was assessed in Werner-deficient (WS) cells stably expressing the wild-type WRN (WRN^WT^), its ATM-unphosphorylable (S > A) mutants (WRN^S1058A^ and WRN^S1141A^), phosphomimetic (S > D) mutants (WRN^S1058D^ and WRN^S1141D^) and, as a control, the end resection-defective WRN mutant (WRN^S1133A^) (see Supplementary Figure S4 for expression levels). We previously demonstrated that formation of ssDNA dependent on S1133 phosphorylation of WRN occurs at 90 min of CPT treatment (20). Loss of CDK1-dependent phosphorylation of WRN reduced ssDNA formation upon CPT exposure, as expected (Figure 2B). Mutation of the S1292 site of WRN (Supplementary Figure S5) as well as expression of the phosphomimetic S1058D-WRN mutant were functionally irrelevant for end resection (Figure 2B). However, expression of the S1058A-WRN mutant significantly impaired end resection compared to the wild-type WRN (Figure 2B and C).

In sharp contrast with the S1058A-WRN mutant, the S1141A-WRN mutant was end resection proficient and, at the early time-point after CPT treatment, showed a slightly enhanced level of ssDNA compared to the wild-type WRN (Figure 2C). The defective end resection phenotype of the S1058A-WRN mutant was induced also by treatment with the radiomimetic drug Bleomycin, which did not affect the S1141A-WRN mutant phenotype (Supplementary Figure S6A). Furthermore, as evidenced by the IdU/ssDNA assay, all the mutants affecting end resection after fork collapse failed to induce any alteration in ssDNA exposure after replication arrest (Supplementary Figure S6B). Collectively, these results suggest that phosphorylation at these sites is not involved in regulating degradation of intact forks, even if assessed by the ssDNA analysis and not also by the DNA fibre assay, but is important for processing DSBs.

**Figure 3.**
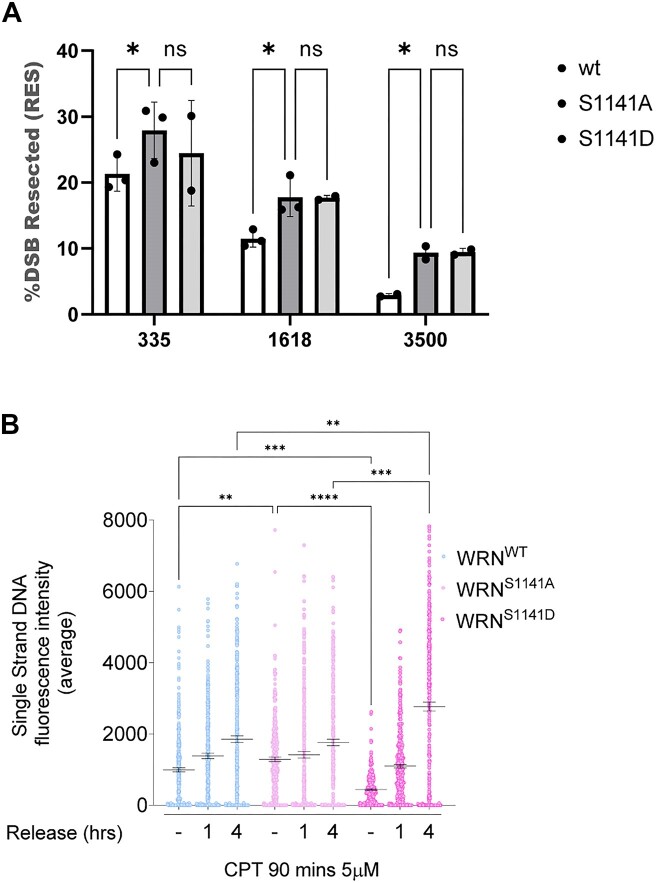
Altered phosphorylation of WRN at S1141 affects end resection. (**A**) The graph shows the analysis and quantification of Cas9-induced and processed DSBs in WS fibroblasts transiently transfected with WRN mutants. Data are represented as the percentage of DSBs resected on a specific chromosomal site. (**B**) WS-derived cell lines complemented with different WRN mutants were labelled and treated with CPT, followed by different time points of release in free medium before performing IdU/ssDNA assay. The dot plot shows the mean intensity of IdU/ssDNA staining for single nuclei (*n* = 300, two biological replicates). Data are presented as mean ± SE. Statistical analysis was performed by the ANOVA test (****P< 0.0001, **P< 0.01, *P> 0.01; ns = not significant).

Cells expressing the phosphomimetic S1141D-WRN mutant apparently showed reduced end resection after CPT treatment. To further investigate the phenotype exhibited by the S1141-WRN mutants, we performed a quantitative evaluation of end resection at single DSB sites introduced by Cas9/sgRNA (29). WS cells were transiently transfected with plasmids expressing the wild-type WRN or its S1141A mutant, along with plasmids expressing Flag-Cas9 and sgRNA guides against one AsiSI site (Supplementary Figure S7). Formation of ssDNA at Cas9-induced DSB was analysed by droplet-digital PCR (ddPCR) at increasing distances from the cutting site and normalized against the cut efficiency (29). Although loss of Ser1141 phosphorylation of WRN increased the number of resected ends at each of the distances analysed, the effect was more evident at the most distant site (Figure 3A). Unexpectedly, considering the reduced detection of ssDNA in the ssDNA/IdU assay (Figure 2C), the phosphomimetic S1141D-WRN mutant showed increased levels of ssDNA in the Cas9/sgRNA resection assay (Figure 3A). The collected data, in conjunction with the ssDNA/IdU assay, indicate that the deregulated phosphorylation of WRN at Ser1141 influences the end resection process.

However, the impact differs significantly when compared to the effects of Ser1133 or Ser1058. One possible explanation for the conflicting results on the end resection phenotype associated with S1141D mutation of WRN lies in the treatment timing. The ssDNA/IdU assay was performed very early during CPT treatment, which is close to the induction of DSBs. In contrast, the Cas9/sgRNA assay has a wider kinetics window and includes analysis of a later stage during DSBs repair. Thus, we evaluated formation of ssDNA at the nascent strand during recovery from CPT treatment. Formation of ssDNA was analysed by the IdU/ssDNA assay at 0, 1 and 4 h of recovery from 90-min CPT treatment (Figure 3B). In wild-type cells, formation of nascent ssDNA was detected also during recovery and increased with time. Cells expressing the S1141A-WRN mutant showed enhanced formation of ssDNA compared to the wild-type during CPT treatment, but the ssDNA level remained stable with time during recovery. Of note, this ssDNA was still dependent on resection as the combination of the S1141A mutation with the end resection-defective S1133A (2A), suppressed its accumulation (Supplementary Figure S8). Slightly reduced levels of ssDNA were detected in the S1141D-WRN mutant during treatment, while a striking increase was observed during recovery, recapitulating what was observed in the Cas9/sgRNA assay.

To determine whether the observed increase in ssDNA exposure in S1141D-WRN cells during recovery from CPT was dependent on end resection-related nucleases, we performed the IdU/ssDNA assay on cells expressing either the wild-type WRN or the S1141D-WRN mutant previously transfected with EXO1 siRNA or treated with DNA2 inhibitor during the recovery period. Formation of nascent ssDNA in both wild-type and S1141D-WRN was dependent on DNA2 and EXO1, albeit to varying degrees (Supplementary Figure S9). Notably, the ssDNA observed in the S1141D-WRN mutant during recovery was significantly more sensitive to DNA2 inhibitor compared to the wild-type.

Overall, these findings suggest that phosphorylation of WRN at Ser1058 by ATM, together with that performed by CDK1 at Ser1133, is critical for end resection, while the phosphorylation status of Ser1141 might be relevant for limiting or avoiding aberrant resection originating during recovery from CPT-induced DNA damage.

### Ser1141 and Ser1133 of WRN are inversely regulated during distinct stages of HR

**Figure 4.**
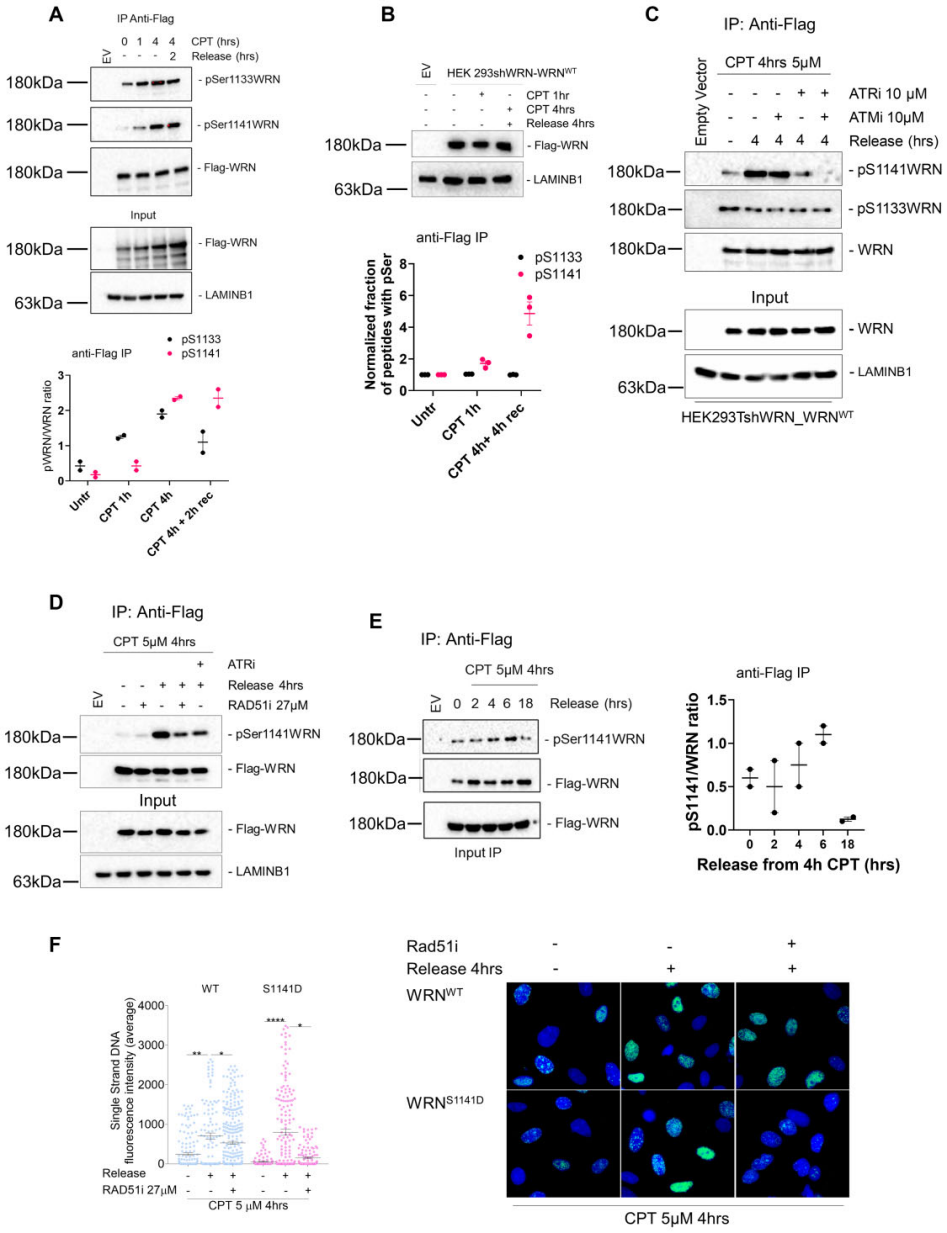
Phosphorylation of WRN by ATM/ATR occurs at the end of resection and requires its correct execution. (**A**) Cells were transiently transfected with an empty vector or a vector expressing Flag-tagged WRN wild-type (WRN^WT^) and were treated with CPT for different time points. In addition, treated cells were recovered for 2 h in drug-free medium. Flag-WRN was immunoprecipitated and 9/10 of IPs were analysed by WB with both the anti-pS1133WRN and the anti-pS1141WRN antibodies, while 1/10 was detected by anti-WRN. One-fiftieth of the lysate was blotted with an anti-Flag antibody to verify transfection. An anti-LaminB1 antibody was used as a loading control. The graph shows the kinetics of WRN phosphorylation levels, and data are from biological duplicates. (**B**) Cells were transiently transfected as in (A). Cells were treated with CPT for 4 h and recovered in CPT-free medium. Flag-WRN was immunoprecipitated, and IPs were analysed by mass spectrometry. The blot shows the amount of WRN from the IP of one representative triplicate. The graph represents quantification of the normalized amounts of phosphorylated residues from biological triplicates. (**C**) Cells were transiently transfected as in (A). Cells were treated with CPT for 4 h. Cells were recovered in CPT-free medium and treated with ATMi (KU-55933), ATRi (VE-821), alone or in combination. Flag-WRN was immunoprecipitated and IPs were analysed and blotted as in (A). (**D**) Cells were transiently transfected as in (A). Cells were treated with CPT in combination or not with the inhibitor and recovered in drug-free medium. Flag-WRN was immunoprecipitated and IPs were analysed and blotted as in (C). (**E**) Cells were transiently transfected as in (A). Flag-WRN was immunoprecipitated and IPs were analysed and blotted as in (C). (**F**) WS-derived cell lines complemented with different WRN mutants were labelled, treated with CPT, and IdU/ssDNA assay was performed. The graph shows the mean intensity of IdU/ssDNA staining for single nuclei measured from three independent experiments (*n*= 300, each biological replicate). Data are presented as mean ± SE. Representative images of IdU/ssDNA-stained cells are shown. Statistical analysis was performed by the ANOVA test (****P< 0.0001, **P< 0.01, *P> 0.05).

We found that phosphorylation of WRN at Ser1141 may be involved in controlling the resection of DSBs during recovery from CPT-induced DNA damage (i.e. when DSBs are already resected, and the repair stage of HR takes place). Thus, modification of Ser1141 and Ser1133 could be differently regulated during these separate stages of HR. To test this hypothesis, we performed IP/WB to evaluate Ser1133 or Ser1141 phosphorylation at 1 and 4 h of treatment (Figure 4A), which correspond to end resection stage and initial recruitment of RAD51, respectively, or after recovery when DSBs are repaired (Supplementary Figure S10A and B; (20)). Phosphorylation at Ser1133 was easily detectable even in untreated conditions, and consistent with its role during end resection, it was detectable already at 1 h of treatment and remained elevated at 4 h (Figure 4A). However, phosphorylation at Ser1133 of WRN somewhat declined after recovery (Figure 4A), when formation of RAD51 foci increased strongly (Supplementary Figure S10B). In contrast, phosphorylation at Ser1141 was barely detectable in untreated conditions and at 1 h of treatment but increased strongly at 4 h and remained elevated also during recovery from CPT (Figure 4A and Supplementary Figure S11). This behaviour was also observed in the endogenous WRN immunoprecipitated from an HEK293T-derived cell line that has been engineered to express a doxycycline-inducible shWRN cassette (Supplementary Figure S12). To provide further confirmation of this different kinetics behaviour by an independent approach, we performed relative quantification of the phosphorylation at Ser1133 and S1141 by mass spectrometry (Figure 4B and Supplementary Figure S13). Quantitative analysis of phosphorylation at the two residues confirmed the trend detected by IP/WB and evidenced that a consistent fraction of WRN molecules bears Ser1133 phosphorylated already in untreated conditions.

Engagement of the post-resection stage of HR has been correlated with ATR-dependent phosphorylation (30), and Ser1141 is phosphorylated by ATR during prolonged recovery from CPT treatment (31). Consequently, we asked if increased phosphorylation of WRN at Ser1141 shortly after recovery from CPT could depend on ATR. Thus, we analysed the status of Ser1141 in cells after 4 h of recovery from treatment with CPT and ATR and/or ATM inhibitors, as well as the level of Ser1133 phosphorylation, a readout of the pro-resection role of WRN (Figure 4C; (20)). As expected, phosphorylation of Ser1141 increased during recovery, while phosphorylation of Ser1133 was elevated during treatment but decreased thereafter (Figure 4C). Surprisingly, phosphorylation of Ser1141 was barely affected by ATM inhibition but drastically reduced by ATR inhibition (Figure 4C). This suggests that inhibition of ATM affects the level of pSer1141 only indirectly because of its interference with end resection initiation, which is an upstream event in the subsequent ATR-dependent targeting of Ser1141. Moreover, ATR-dependent phosphorylation at Ser1141 was impaired in cells expressing the unphosphorylable S1133A-WRN mutant, which impairs long-range end resection (20), but not in wild-type cells in which ATM inhibitor was added during recovery (Supplementary Figure S14). These results indicate that the two phosphorylation events define distinct molecular switches for the role of WRN during the resection or the repair stage by HR.

To determine whether phosphorylation of WRN at Ser1141 could occur after the initiation of strand-invasion, we repressed the formation of RAD51 nucleofilaments with the B02 compound (RAD51i) and analysed the level of WRN phosphorylation by IP/WB in CPT and during recovery (Figure 4D). As already observed, Ser1141 phosphorylation of WRN was low during treatment and not affected by RAD51 inhibition. However, during recovery, RAD51 inhibition greatly reduced Ser1141 phosphorylation, suggesting that this modification depends on strand invasion. To confirm this observation, we depleted RAD51 by RNAi in HEK293TshWRN cells before adding-back Flag-WRN and evaluated Ser1141 phosphorylation by IP/WB. Depletion of RAD51 reduced the level of Ser1141 phosphorylation during treatment and recovery (Supplementary Figure S15), the latter consistent with inhibition by B02.

Next, we assessed the dephosphorylation of Ser1141 and correlated it with the repair of CPT-induced DSBs. To this purpose, we performed IP/WB assays at various time points following CPT release, spanning from 4 to 18 h. This period covers the repair timeline for the majority, if not all, of the DSBs induced by the 90-min CPT treatment (20). The level of pSer1141 remained elevated throughout recovery but greatly declined after 18 h from CPT (Figure 4E), when most of the DSBs were repaired in wild-type cells (Figure 6 and Supplementary Figure S16A).

Ser1141 phosphorylation occurs downstream of end resection, depends on the RAD51 nucleofilament formation and declines over time during DSBs resealing. Thus, we reasoned that inhibition of RAD51 nucleofilaments, which interferes with strand invasion and prevents Ser1141 phosphorylation (Figure 4D), could reduce the accumulation of ssDNA during recovery. To verify this hypothesis, we analysed nascent ssDNA in cells treated with a RAD51 inhibitor during recovery from CPT (Figure 4F). RAD51 inhibition only slightly reduced nascent DNA levels in wild-type cells but strongly suppressed them in the S1141D-WRN mutant.

Collectively, these data indicate that phosphorylation at Ser1133 and Ser1141 of WRN are inversely regulated and suggest that ATR-dependent modification of Ser1141 occurs immediately lately after long-range end resection, increases during strand-invasion, and then needs to be turned off to avoid excessive accumulation of RAD51-dependent ssDNA.

### RAD51 foci formation and repair by HR require regulated phosphorylation of WRN at Ser1141

**Figure 5.**
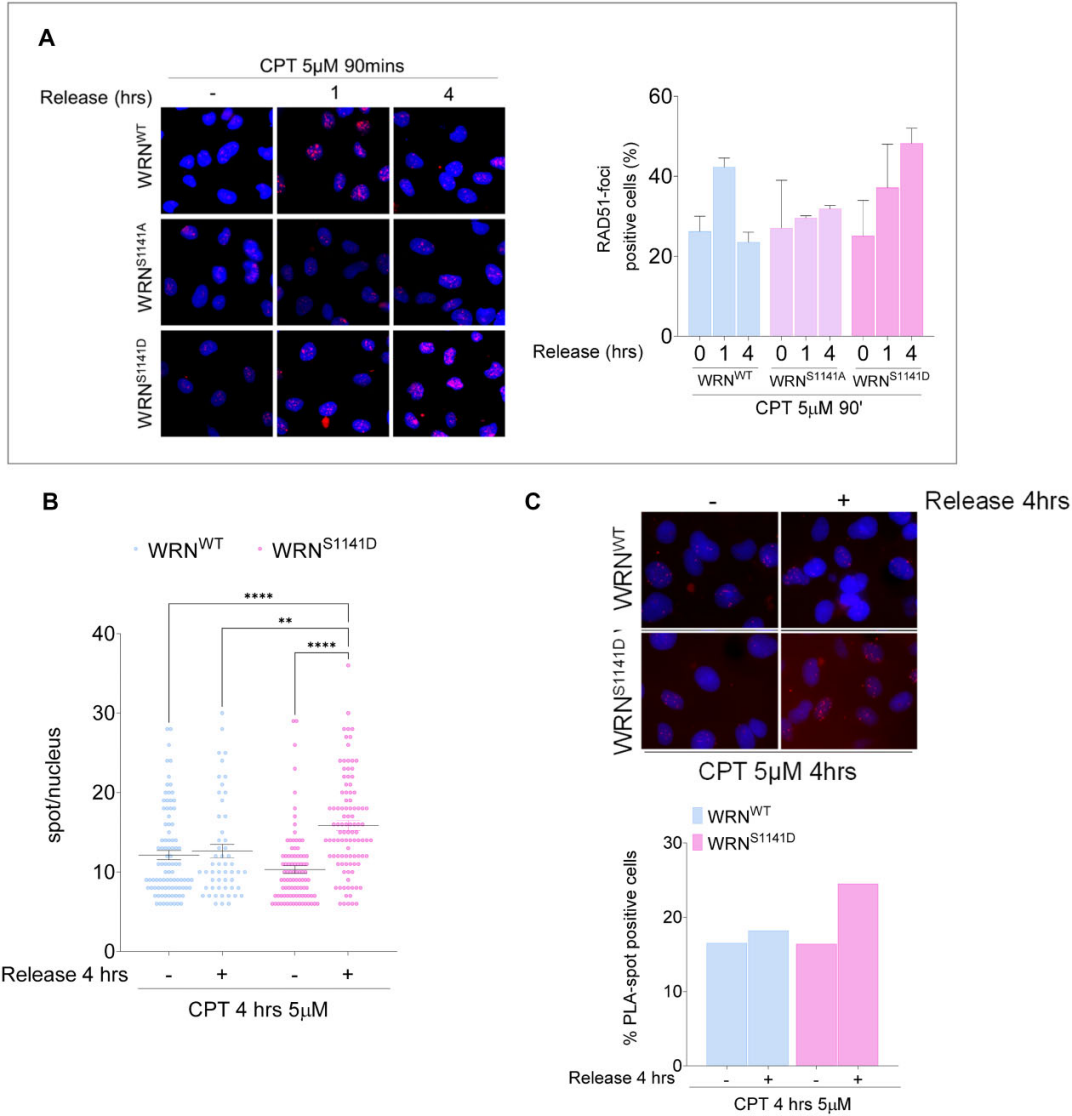
Regulated phosphorylation of WRN at S1141 is involved in RAD51 localization. (**A**) Cells stably expressing Flag-tagged WRN mutants were treated with CPT and allowed to recover for different time points as indicated. RAD51-foci staining was then analysed by IF. The graph shows the percentage of RAD51-foci positive cells measured from two independent experiments (*n* = 200, each biological replicate). Data are presented as mea ± SE. Representative images of RAD51 staining in response to treatment are shown. (**B**) Analysis of ssDNA/RAD51 interaction by *in situ* PLA. Cells were treated with CPT and allowed to recover for 4 h, then subjected to PLA using anti-IdU and anti-RAD51 antibodies. The panels show representative PLA images showing association of ssDNA with RAD51 (scale bar: 10 μm). The dot plot shows the PLA spots per PLA positive cells. At least 100 nuclei were analysed for each experimental point (*n* = 2). Values are presented as mean ± SE. Statistical analysis was performed by the ANOVA test (****P< 0.0001, **P< 0.01, each biological replicate). (**C**) The graph shows the number of PLA positive cells. At least 100 nuclei were analysed for each experimental point. Representative images from neutral Comet assay are shown.

Having demonstrated that phosphorylation of WRN by ATR at Ser1141 involves RAD51 nucleofilament formation, we examined whether deregulated phosphorylation at this site affected RAD51 foci formation after recovery from CPT treatment (Figure 5A). Wild-type cells showed increased RAD51 foci at 1 h of recovery, which decreased thereafter. Consistent with phosphorylation occurring during recovery, no significant differences were observed in the ability to form RAD51 foci during the short CPT treatment in cells expressing the S1141-WRN mutants compared to wild-type cells (Figure 5A). The inability to phosphorylate WRN at Ser1141 (S1141A-WRN mutant) altered the kinetics of RAD51 foci formation. By contrast, the number of RAD51-positive cells and the brightness of foci were greatly increased and sustained in the S1141D-WRN mutant during recovery.

Since RAD51 is loaded onto nascent ssDNA at collapsed replication forks but also onto parental ssDNA at stalled forks, we evaluated the presence of RAD51 at nascent ssDNA by PLA to confirm that recruitment of RAD51 occurred at collapsed forks (32). Our results showed that RAD51 was associated with nascent ssDNA in wild-type cells during recovery from CPT treatment and, consistent with IF analysis, this association was increased in the S1141D-WRN mutant (Figure 5B and C).

**Figure 6.**
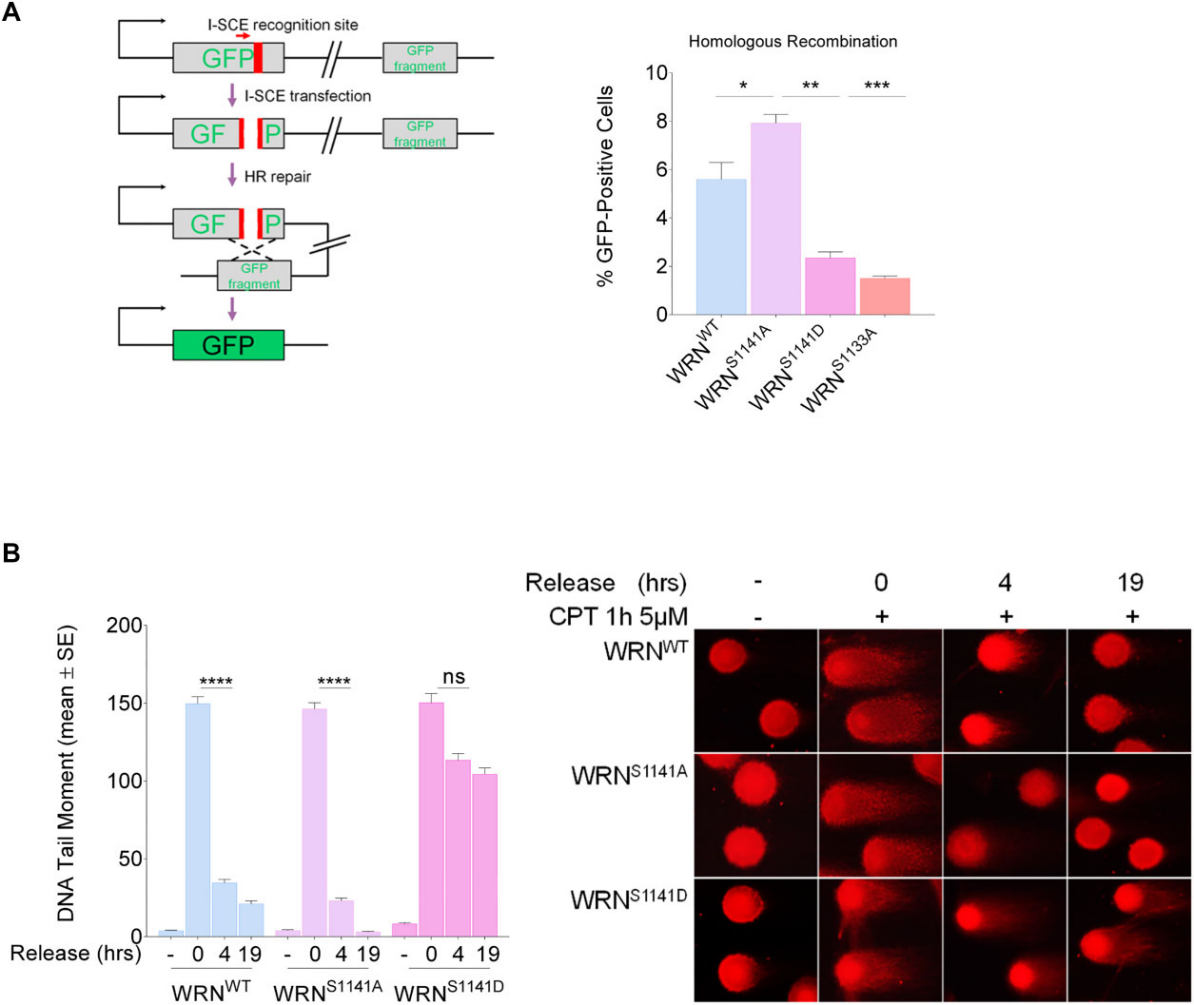
Regulated phosphorylation of WRN at S1141 is essential for DSB repair. (**A**) Analysis of efficiency of HR-mediated repair by reporter assay. HEK293TshWRN cells were co-transfected with the indicated WRN forms, the I-SceI expression vector pCBASce and the pDRGFP HR reporter plasmid, as described in ‘Materials and Methods’ section. The graph shows the percentage of GFP positive cells measured by flow cytometry. Data are presented as mean ± SE from three independent experiments (ns = not significant; *P< 0.05, **P< 0.01, ***P< 0.001; ANOVA test; *n*= 3 × 10^5^ events each biological repeat). (**B**) DSB repair efficiency analysis. WS-derived SV40-trasformed fibroblasts stably expressed Flag-tagged WRN mutants were treated with CPT for 1 h and then release in drug-free medium at different time points. DSB repair was evaluated by the neutral Comet assay. In the graph, data are presented as mean tail moment ± SE from three independent experiments. Representative images from the neutral Comet assay are shown. Statistical analysis was performed by the ANOVA test (****P< 0.0001; ns = not significant, each biological replicate).

To verify whether high levels of RAD51 foci represented unproductive recombination events leading to defective HR, we next examined the ability of WRN mutant cells to perform RAD51-dependent repair by gene conversion using a reporter assays (Figure 6A) (20,33). The pDRGFP HR reporter was transiently transfected into HEK293TshWRN cells along with plasmids expressing the wild-type WRN, S1141A-WRN or S1141D-WRN mutant, and the I-SceI endonuclease. As an internal control, we analysed HR efficiency in cells expressing the S1133A-WRN mutant, which is defective in resection and HR (20). At 72 h post-transfection, the number of GFP-positive cells was evaluated by flow cytometry to assess their repair capacity. The ability to repair by HR of S1141A-WRN mutant was comparable to that of wild-type cells, whereas it was compromised in cells expressing S1141D-WRN or S1133A-WRN mutant (Figure 6A).

Next, we examined the relevance of phosphorylation of WRN at Ser1141 on the ability to repair CPT-induced DSBs. We evaluated DSB repair by neutral Comet assay during recovery from a short CPT treatment (Figure 6B). As expected, WRN^WT^ cells almost completely repaired DSBs at 4 h of recovery (Supplementary Figure S16A) In contrast, expression of the S1058A-WRN mutant interfered with HR-dependent DSB repair (Supplementary Figure S16B), similar to what was previously observed in the S1133A-WRN mutant (20). Abrogation of S1141 phosphorylation (WRN^S1141A^) did not have any significant effect except for more efficient repair at 19 h of recovery (Supplementary Figure S16C). Confirming that HR is the primarily repair pathway engaged, DSBs were greatly reduced by RAD51 inhibition but not by DNA-PK or LigI/III inhibition, which block NHEJ or alt-EJ, respectively (Supplementary Figure S16A and C). Conversely, DNA repair was compromised in WRN^S1141D^ cells and only a minor fraction of DSBs was repaired at 19 h of recovery. This residual repair appeared RAD51-dependent (Supplementary Figure S16D) and was rescued by abrogation of end resection through depletion of BRCA1, which redirects DSB repair from HR to NHEJ (Supplementary Figure S17A and B).

Therefore, these findings indicate that switching off ATR-dependent phosphorylation of WRN at Ser1141 is required to promote a correct post-synaptic RAD51 foci formation, preventing aberrant accumulation of RAD51 foci and ensuring repair by HR.

### A switch from CDK1- to ATR-dependent phosphorylation of WRN is required for correct DSB repair by HR

**Figure 7.**
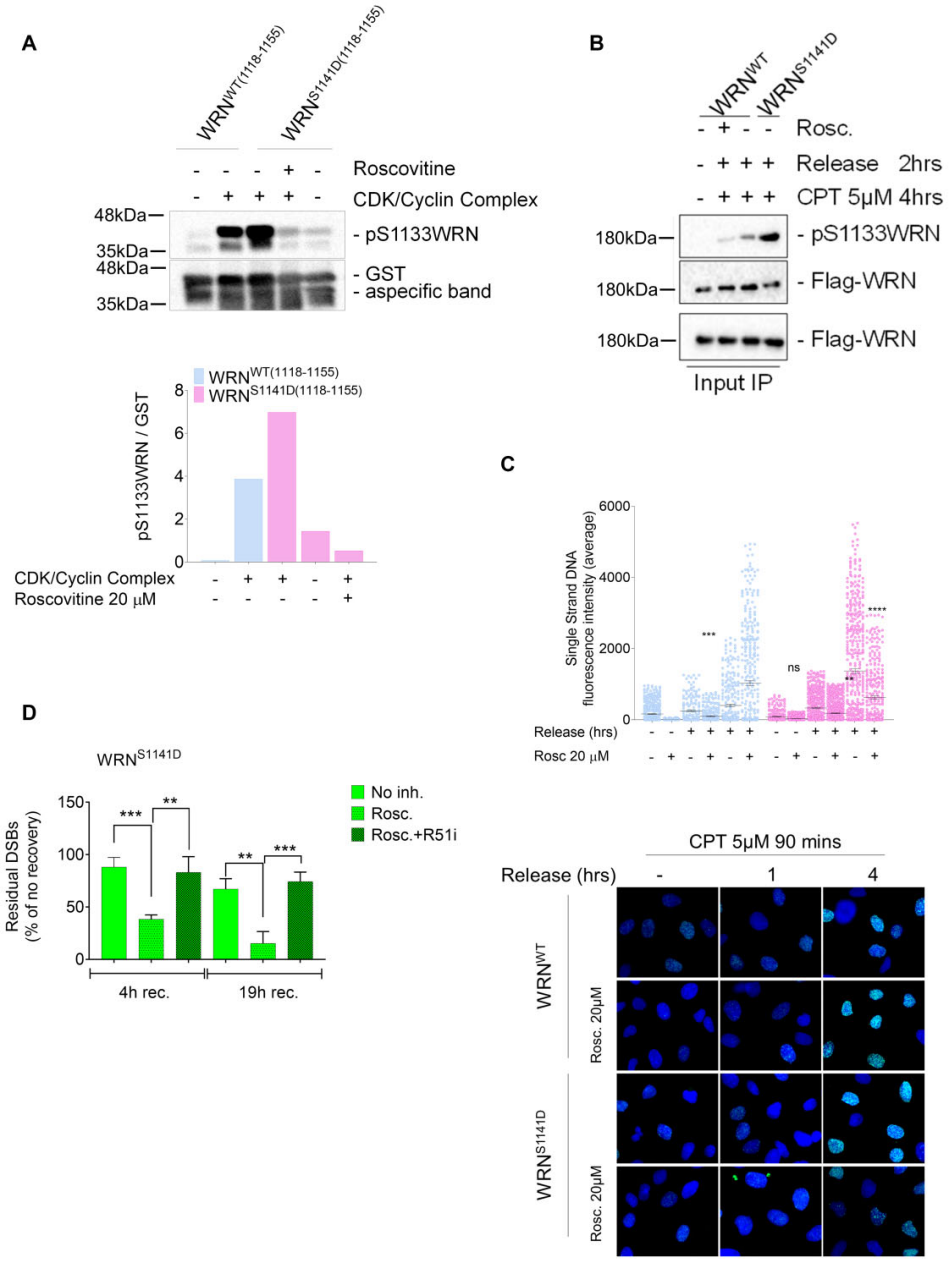
Persisting phosphorylation of WRN at S1141 leads to aberrant modification of S1131 by CDK. (**A**) *In vitro* kinase assay. Approximately 2 μg of immunopurified GST-tagged WRN wild-type fragment (C-WRN^WT^) or WRN phosphomimetic mutant fragment (C-WRN^S1141D^) were phosphorylated *in vitro* using CDK/Cyclin complex, and the experiment was performed treated with CDKi (Roscovitine). Immunoblotting was used to analyse WRN phosphorylation levels in different WRN fragments using an anti-pS1133WRN antibody. The graph shows the pS1133WRN level in each experimental point. (**B**) WRN was immunoprecipitated from cells transiently transfected with Flag-WRN wild-type (WRN^WT^) or its phosphomimetic mutant and treated CPT for 4 h, followed by a 2-h release treated with CDKi (Roscovitine). Nine-tenths of IPs were analysed by western blotting (WB) with the anti-pS1133WRN antibody, while 1/10 was analysed by anti-Flag antibody. Input represents 1/50 of the lysate. Anti-Flag antibody was used to verify transfection. (**C**) WS-derived cell lines complemented with different WRN mutants were labelled, treated with CPT and the IdU/ssDNA assay was performed. The dot plot shows the mean intensity of IdU/ssDNA staining for single nuclei measured from three independent experiments (*n*= 300, each biological replicate). Data are presented as mean ± SE. Representative images of IdU/ssDNA-stained cells are shown. Statistical analysis was performed by ANOVA test (****P< 0.0001, ***P< 0.001; ns = not significant; *n*= 300, each biological replicate). (**D**) WS-derived SV40-trasformed fibroblasts were complemented with phosphomimetic WRN mutant, treated with CPT for 1 h and allowed to recover in the presence or not of the different inhibitors as indicated. The presence of DSBs was evaluated by the neutral Comet assay. In the graph, data are presented as percent of residual DSBs from three independent experiments normalized against the value of the 0 h recovery. Statistical analysis was performed by ANOVA test (***P< 0.001, **P< 0.01, *n*= 300, each biological replicate).

Our findings suggest that ATR-dependent phosphorylation of WRN at Ser1141 must occur at within a specific window of opportunity to promote proper RAD51 foci formation and repair by HR. Persistent or untimely modification of Ser1141, as mimicked by the phosphomimetic S1141D-WRN mutant, leads to aberrant exposure of ssDNA and defective repair of DSBs. Since CDK1 and ATR-dependent phosphorylation of WRN are ordered events, we reasoned that persistence of the phosphomimetic mutation at Ser1141 might promote untimely phosphorylation at the adjacent Ser1133 when this modification should be switched off. To this purpose, we tested by an *in vitro* kinase assay whether phosphorylation at Ser1141 could induce modification at Ser1133. A small fragment of WRN containing the wild-type Ser1141 residue or the S > D phosphomimetic mutation was used as a substrate for CDK2/Cyclin complex. Consistent with our previous data (20), the CDK2/Cyclin complex efficiently phosphorylated WRN at Ser1133 and this phosphorylation was greatly stimulated by the S1141D-WRN mutation (Figure 7A). This suggests that constitutive phosphorylation of Ser1141 disrupts the ordered modifications of WRN in response to DSBs, promoting modification of Ser1133 even when CDK1 activity levels are low and both phosphorylations should be switched off. Thus, we monitored the phosphorylation of Ser1133 by IP/WB in cells expressing the wild-type or the S1141D-WRN protein during CPT treatment or after recovery (Figure 7B). As expected, Ser1133 phosphorylation prevailed during treatment but was reduced at 2 h of recovery. By contrast, mimicking a constitutive phosphorylation at Ser1141 led to sustained Ser1133 modification during recovery.

To determine if concomitant phosphorylation of WRN at Ser1133 and Ser1141 could be responsible for the abnormal exposure of nascent ssDNA observed at 4 h of recovery in the S1141D-WRN mutant, we treated cells with CPT and CDK inhibitor during recovery (Figure 7C). The IdU/ssDNA assay revealed that, in wild-type cells, inhibition of CDK activity increased ssDNA formation, while it largely suppressed the ssDNA accumulated in cells expressing the S1141D-WRN mutant.

To verify whether inhibition of CDKs could restore repair of CPT-induced DSBs in cells expressing the S1141D-WRN mutant, we performed neutral Comet analysis, recovering cells with or without Roscovitine (Figure 7D). Inhibition of CDK activity significantly reduced the level of unrepaired DSBs detected in cells at 4 h after CPT treatment, restoring their RAD51-dependent repair capability.

Collectively, these results demonstrate that aberrant metabolism of CPT-induced DSBs and their deficient repair depend on unscheduled CDK-dependent phosphorylation of WRN at Ser1133 in the presence of a mutation mimicking constitutive ATR-dependent modification at Ser1141.

## Discussion

Here, using regulation-defective mutants, we demonstrate that WRN is critical for the correct execution of long-range end resection and for the metabolism of RAD51 nucleofilaments. WRN undergoes ordered and hierarchical phosphorylation by CDK1, ATM and ATR. Each of these events specifically regulates a defined stage during the repair of DSBs at replication forks, acting as a molecular switch. Unscheduled phosphorylation of WRN by ATR at Ser1141 leads to the abnormal accumulation of ssDNA and the persistence of unproductive RAD51 foci formation, resulting in unrepaired DNA damage.

### CDK1 and ATM cross-talk regulates WRN function during long-range end resection

End resection of DSBs is a highly regulated process requiring the CDK1 activity (13,34). Consistently, WRN is phosphorylated by CDK1 to carry out its pro-resection function (20). However, ATM-dependent phosphorylation of several proteins is also crucial for end resection (13). Notably, WRN is phosphorylated by ATM on multiple residues in response to replication stress or ionizing radiation (21,22). In this study, we show that only two S/TQ residues of WRN, Ser1058 and Ser1141, are important for DSB repair by recombination, but they have distinct functional roles. ATM-dependent phosphorylation of WRN at Ser1058 is essential for long-range end resection and requires prior phosphorylation at Ser1133 by CDK1. Both events implicate ATM and MRE11 nuclease activity, which are involved in initiating end resection (25,35–37). Therefore, our findings support the possibility that WRN phosphorylation occurs only if end resection initiates, and are consistent with the observed requirement of WRN helicase activity in the long-range phase of resection (20,27). It will also be of interest to ascertain the enzymatic activities of the WRN mutants, although we already reported that the S1133A mutant retains normal activity (20). These observations, along with the enhanced ability of ATM to phosphorylate the S/TQ sites of WRN *in vitro* in the presence of the S1133D mutation, suggest that the primary function of Ser1133 phosphorylation is to promote subsequent ATM-dependent phosphorylation at Ser1058.

Additionally, abrogation of Ser1141 phosphorylation of WRN enhances resection at DSBs and delays RAD51 recruitment. Notably, deregulated phosphorylation of EXO1, another nuclease involved in end resection, can also interfere with resection (36,38), suggesting that phosphorylation by ATM or ATR is a common mechanism for regulating end resection.

### Phosphorylation of WRN at Ser1141 and resolution of recombination intermediates

Loss of Ser1141 phosphorylation does not confer a severe hyper-resection phenotype in response to CPT treatment. Interestingly, while expression of a mutation mimicking constitutive phosphorylation at Ser1141 induces mild suppression of ssDNA during CPT treatment, it results in a striking increase in end resection and elevated RAD51 foci late during recovery from CPT. This indicates that Ser1141 phosphorylation needs to be strictly regulated. The remarkable enhancement of ssDNA is driven by nucleolytic processing but depends on strand invasion, as it is repressed by the RAD51 inhibitor B02, which interferes with nucleofilament formation (39).

Replication-dependent DSBs are one-ended and are normally repaired by break-induced replication (BIR) (40). One way replication proceeds by BIR is through the migration of a D-loop (40). If migrating D-loops are unproductive, they lead to strand rejection and extensive resection far from the break site (41). In yeast, Srs2 strippase and helicase activities can resolve RAD51 intermediates during BIR, preventing accumulation of unproductive intermediates and ssDNA behind the migrating D-loop (42). Although WRN does not have RAD51 strippase activity, it can process D-loops *in vitro* (18), and deregulated phosphorylation at Ser1141 could interfere with this function. Dephosphorylated WRN at Ser1141 may dismantle unproductive RAD51 filaments, as seen with the related helicases BLM and RECQ5 (43,44). However, expression of the phosphomimetic S1141D-WRN does not induce a hyper-recombination phenotype but rather decreases recombination efficiency, arguing against this hypothesis. Moreover, the inability of S1141D-WRN to repair CPT-induced DSBs is due to the accumulation of unproductive RAD51 filaments and ssDNA, which blocks other DNA repair activities. Consistent with this hypothesis, the DSB repair defect in cells expressing S1141D-WRN can be mitigated by BRCA1 depletion and by switching CPT-induced DSB repair from HR to NHEJ.

Interestingly, the aberrant handling of RAD51-dependent intermediates induced by the expression of the S1141D-WRN mutant does not derive directly from the phosphomimicking mutation but rather from the concomitant pathological stimulation of Ser1133 phosphorylation by CDKs. Indeed, both the abnormal accumulation of ssDNA during recovery and the persistent DSBs are reverted if CDKs are inhibited during recovery.

## Conclusions

**Figure 8.**
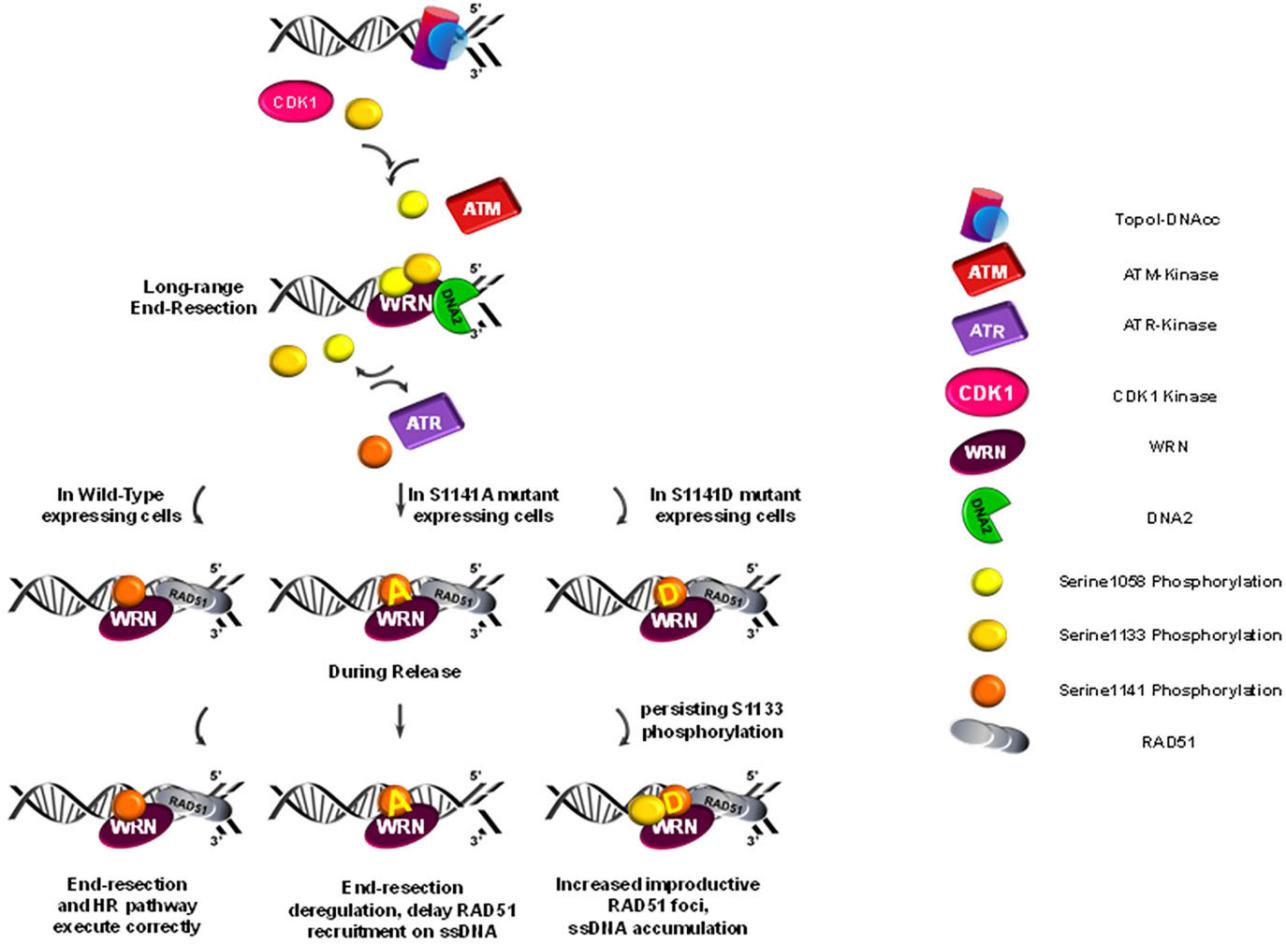
Proposed model of regulation of WRN in the repair of DSBs at the replication fork. A schematic representation illustrating the proposed model of WRN regulation during the repair of DSBs at the replication fork is depicted. The model outlines the sequential phosphorylation events involving CDK1, ATM and ATR, along with their respective target sites on WRN (S1058, S1133 and S1141). It highlights the role of these phosphorylation events in modulating WRN function during different stages of DSB repair, including end resection, RAD51 nucleofilament formation and resolution of recombination intermediates. The model also suggests the importance of timely and ordered phosphorylation of WRN for proper DSB repair by HR. Further details are provided in the accompanying text.

Our observations can be summarized in the model shown in Figure 8. In response to replication-dependent DSBs and after short-range resection, WRN is phosphorylated at Ser1133 by CDK1. This phosphorylation primes the phosphorylation at S1058 by ATM, which is a crucial regulatory event for WRN-DNA2-dependent long-range resection. After long-range resection has started, WRN needs to be dephosphorylated at Ser1133 and probably at Ser1058, while it is targeted by ATR at Ser1141 after RAD51 nucleofilament formation, thus limiting end resection. Subsequently, dephosphorylation of WRN at Ser1141 contributes to proper regulation of RAD51 foci dismantling and/or resolution of the migrating D-loop during HR. This process might be facilitated by protein degradation, as reported earlier (31). Inability to regulate or perform this ordered phosphorylation cascade would result in either an inability to sustain end resection or to repair DSBs by HR. Although we did not used DNA fiber assay to evaluate nascent strand degradation after fork stalling but only formation of ssDNA at nascent strand as an indirect proxy of fork degradation, our data would suggest that phosphorylation at these sites is more relevant after fork collapse.

Using regulatory and separation-of-function mutants of WRN, our study reveals that the response to replication-dependent DSBs relies on multiple roles of WRN and a complex regulatory layer that controls these roles. Hence, our work indicates that WRN may act as a molecular switch to coordinate end resection and the repair stages of HR. Intriguingly and consistent with a key role of WRN in several DNA transactions at replication fork, multiple phosphorylation sites are found in WRN (Figure 1C). These sites are mainly localized in the acid domain between the exonuclease and helicase core or in the C-terminal region, close to the two domains involved in interactions with other proteins or DNA (Figure 1C). We recently found that phosphorylation of some CK2-dependent sites in the acidic domain of WRN is crucial for binding with RPA70 (45). Although the abrogation of these modifications does not affect end resection of CPT-induced DSBs (45), it would be interesting to investigate whether regulatory events at the N- or C-terminal regions of WRN cross-talk. The WRN helicase has been recently implicated in a synthetic lethal relationship with the microsatellite instability in cancer (46). Although the proposed role of WRN helicase in this context is apparently unrelated to HR-mediated repair of DSBs (46), it would be valuable to define the precise regulatory network behind this genetic relationship, especially considering the recent discovery of novel and specific WRN helicase inhibitors (47,48).

## Supplementary Material

gkae807_Supplemental_File

## Data Availability

The data that support the findings of this study are available from the corresponding author upon reasonable request. The row MS data have been deposited to the ProteomeXchange Consortium via the MassIVE partner repository with the MSV000094766 dataset identifier.
