# Supplementary material for: Switch-like phosphorylation of WRN integrates end-resection with RAD51 metabolism at collapsed replication forks
Source: Nucleic Acids Res. 2024 Sep 24;52(20):12334–50. doi: 10.1093/nar/gkae807 (PMC11551760; doi:10.1093/nar/gkae807)
Supplement: gkae807_Supplemental_File [file gkae807_supplemental_file.pdf]

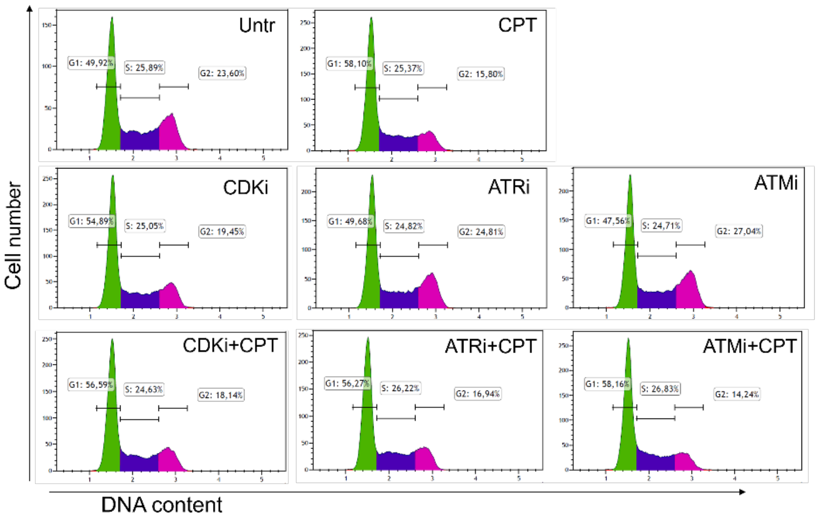

Table 1

Cell cycle progression			
	G_0/G_1 (%)	S-phase (%)	G_2/M (%)
Untr	50.52	24.84	24.64
CDKi	50.41	30.53	19.05
ATRi	51.5	23.5	25
ATMi	44.4	25.38	30.22
CPT	60.07	22.34	17.59
CDKi+CPT	57.95	21.49	20.56
ATRi+CPT	56.91	25	18.09
ATMi+CPT	59.85	24.29	15.85

Supplementary Figure S1. Analysis of cell cycle progression with flow cytometry.

HEK293T cells were treated with CDKi (Roscovitine), ATRi (VE-821) or ATMi (KU-55933) for 30 minutes, then exposed to CPT for 4 hours. Cells were harvested and stained with PI prior FACS analysis. Cell cycle profiles and the Table 1 show the percentages of cells distributed into G0/G1, S or G2/M phases for the representative experiment calculated on the events belonging to these phases only.

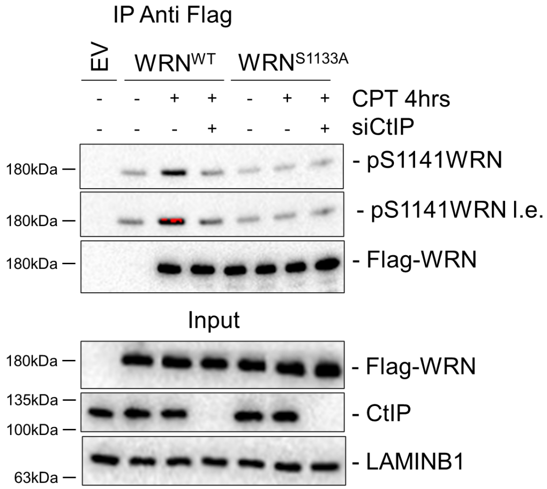

Supplementary Figure S2. Phosphorylation of WRN at Ser1141 requires end resection activity

HEK293TshWRN cells were transfected with siCTRL or siCtIP oligos and plasmid expressing the indicated form of the Flag-WRN protein. After transfection, cultures were treated with CPT for 4 hours. Cells were lysed and WRN protein was immunoprecipitated with anti-Flag-conjugated beads. Nine-tenth of IPs were analysed by WB with the anti-pS1141WRN antibody, while 1/10 was detected by anti-Flag antibody, as indicated. One-fiftieth of the lysate (input) was blotted with an anti-Flag antibody to verify transfection. An anti-LaminB1 antibody was used as loading control.

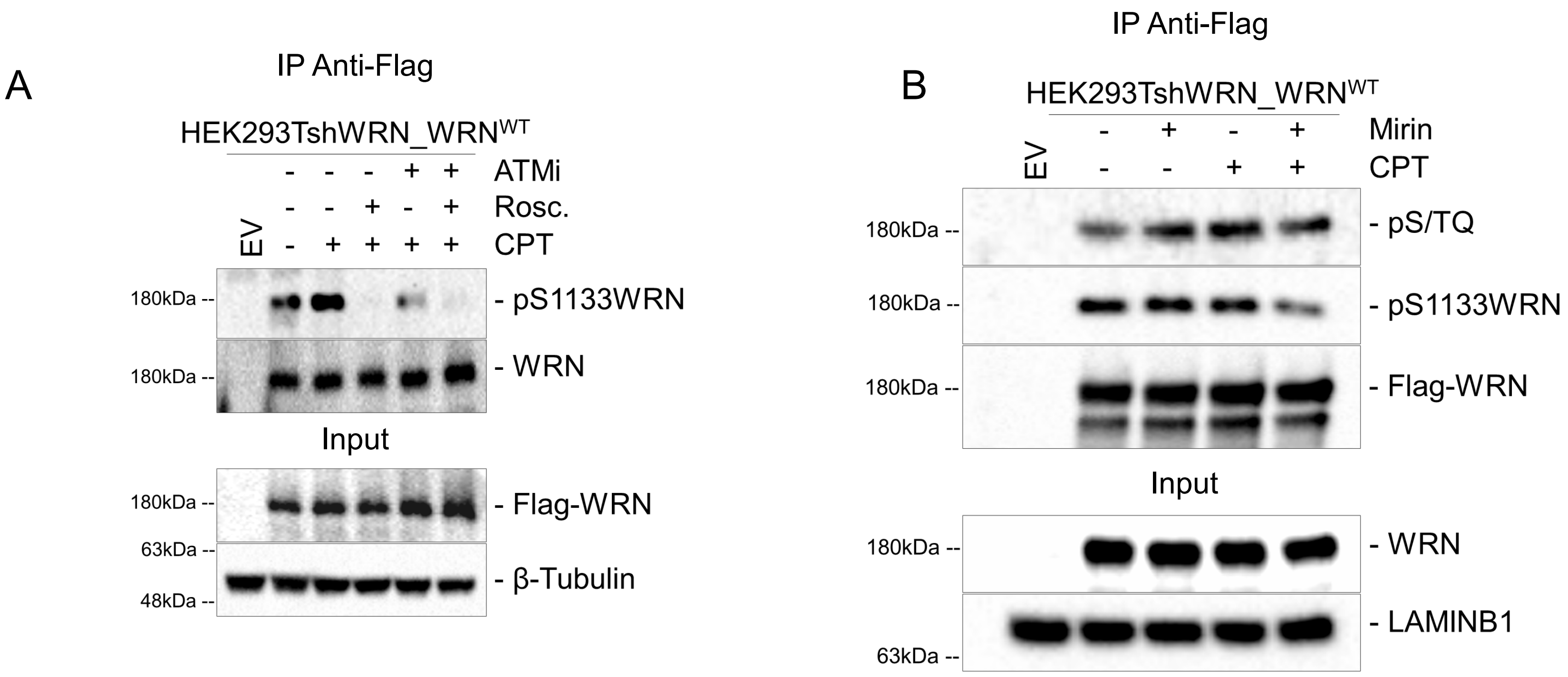

Supplementary Figure S3. Phosphorylation of WRN requires MRE11 nuclease activity

A) Cells were treated with ATMi (KU-55933) or CDKi (Roscovitine) alone or in combination, and with CPT for 4 hours. Cells were lysed and WRN protein was immunoprecipitated with anti-Flag-conjugated beads. Nine-tenth of IPs were analysed by WB with the anti-pS1133WRN antibody, while 1/10 was detected by anti-Flag antibody, as indicated. One-fiftieth of the lysate (input) was blotted with an anti-Flag antibody to verify transfection. An anti-LaminB1 antibody was used as loading control. B) Cells were treated with Mirin and CPT for 4 hours. Cells were lysed and WRN protein was immunoprecipitated with anti-Flag-conjugated beads. Nine-tenth of IPs were analysed by WB with the anti-pS1133WRN antibody and the anti-pS/TQ antibody, while 1/10 was detected by anti-Flag antibody, as indicated. One-fiftieth of the lysate (input) was blotted as in “A”.

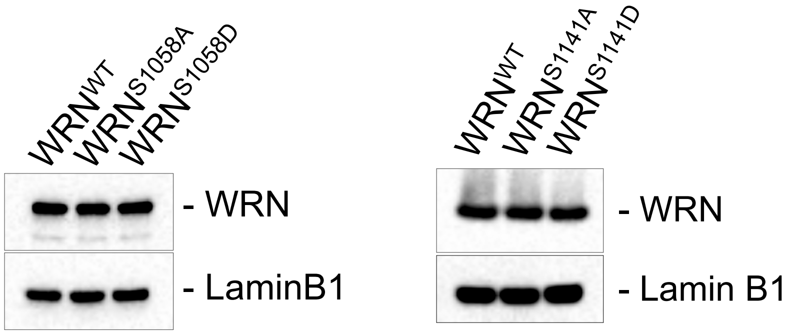

Supplementary Figure S4. Analysis of WRN level

WS-derived SV40-trasformed fibroblasts stably expressing the wild-type form of WRN or the indicated mutant form of WRN were analysed for the protein level by WB using anti-WRN antibody. An anti-LaminB1 was used as a loading control.

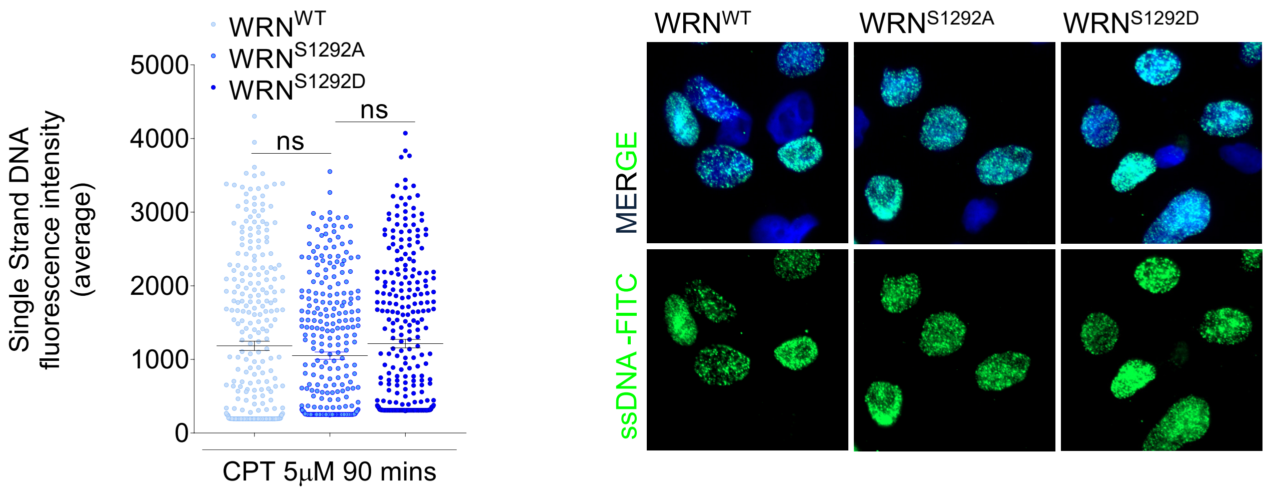

Supplementary Figure S5. Analysis of the relevance of Ser1292 phosphorylation for ssDNA formation in response to CPT

WS-derived SV40-trasformed fibroblasts transiently expressing WRN mutants, as indicated, were labelled, treated and IdU/ssDNA assay was performed. The dot plot shows the mean intensity of ssDNA staining for single nuclei measured from two independent experiments (n=300, each biological replicate), data are presented as mean ± SE. Representative images of IdU/ssDNA-stained from CPT-treated cells are shown. Statistical analysis was performed by the ANOVA test (ns = not significant).

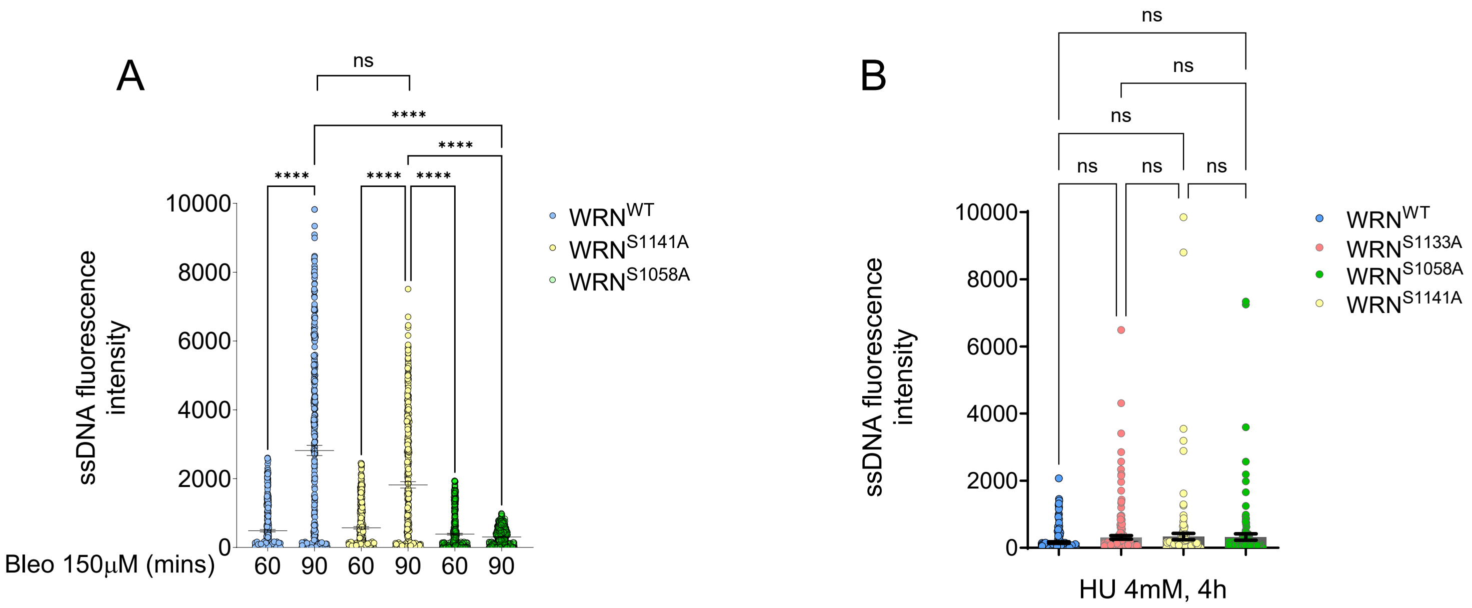

Supplementary Figure S6. Analysis of ssDNA formation after Bleomycin or replication stress

A) WS-derived SV40-trasformed fibroblasts stably expressing the wild-type form of WRN, the S1058A, or S1141A mutant were treated with Bleomycin (Bleo) as indicated. The presence of ssDNA was analysed by non-denaturing IdU/ssDNA assay. The graph shows the mean intensity of IdU/ssDNA staining for single nuclei measured from three independent experiments (at least n=300, minimm 100 each biological replicate), bars represent mean ± SE. Statistical analysis was performed by the ANOVA test (**** = $p<0.0001$). B) WS-derived SV40-trasformed fibroblasts transiently expressing WRN mutants, as indicated, were labelled, treated with HU as indicated and IdU/ssDNA assay was performed. The dot plot shows the mean intensity of ssDNA staining for single nuclei measured from two independent experiments (n=300, 100 each biological replicate), bars are mean ± SE. Statistical analysis was performed by the ANOVA test (ns = not significant).

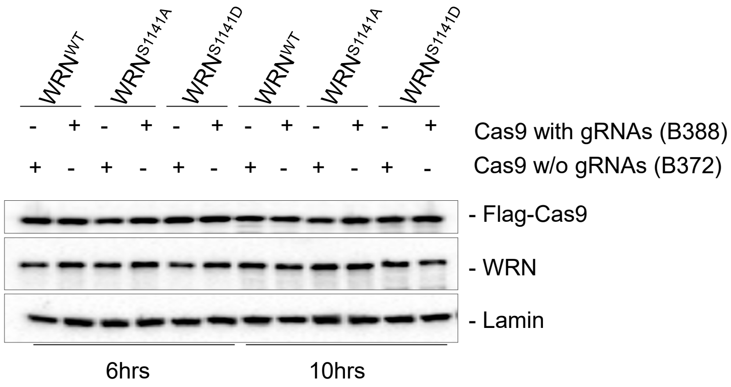

Supplementary Figure S7. Analysis of WRN and Cas9 expression levels with or without guides

WS-derived SV40-trasformed fibroblasts stably expressing the wild-type form of WRN or the indicated mutant form of WRN were transiently transfected with the plasmid expressing Cas9 with or without the indicated sgRNAs. The expression level of Cas9 was analysed by WB using anti-Flag antibody. An anti-WRN antibody was used to analyse WRN expression and anti-LaminB1 was used as a loading control.

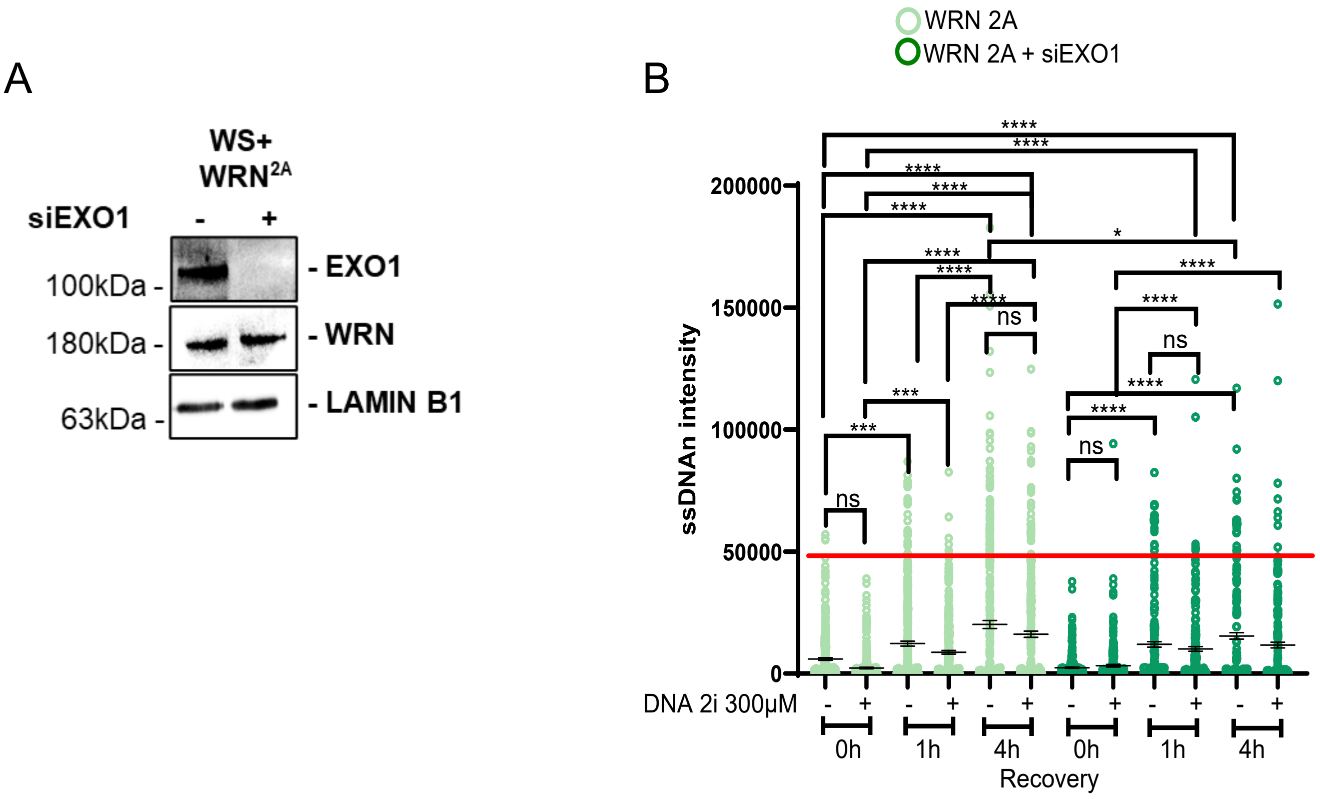

Supplementary Figure S8. Effect of combined S1133A and S1141A mutations on the formation of ssDNA during processing of CPT-induced DSBs

A) WB showing EXO1 depletion in WS-derived SV40-trasformed fibroblasts transfected with the 2A (S1133A+S1141A) mutant form of WRN. B) WS-derived SV40-trasformed fibroblasts expressing the 2A form of WRN, transfected or not with siEXO1 oligos, were treated with 90min CPT in combination or not with DNA2i as indicated. The presence of ssDNA was analysed by non-denaturing IdU/ssDNA assay. The graph shows the mean intensity of IdU/ssDNA staining for single nuclei measured from two independent experiments (n=100, each biological replicate), bars show mean ± SE. The red line indicates the averaged intensity of ssDNA repeatedly detected in cells expressing the wild-type form of WRN. Statistical analysis was performed by the ANOVA test (**** = P<0.0001; *** = p<0.001 ** = P<0.01; * = p<0.05; ns = not significant).

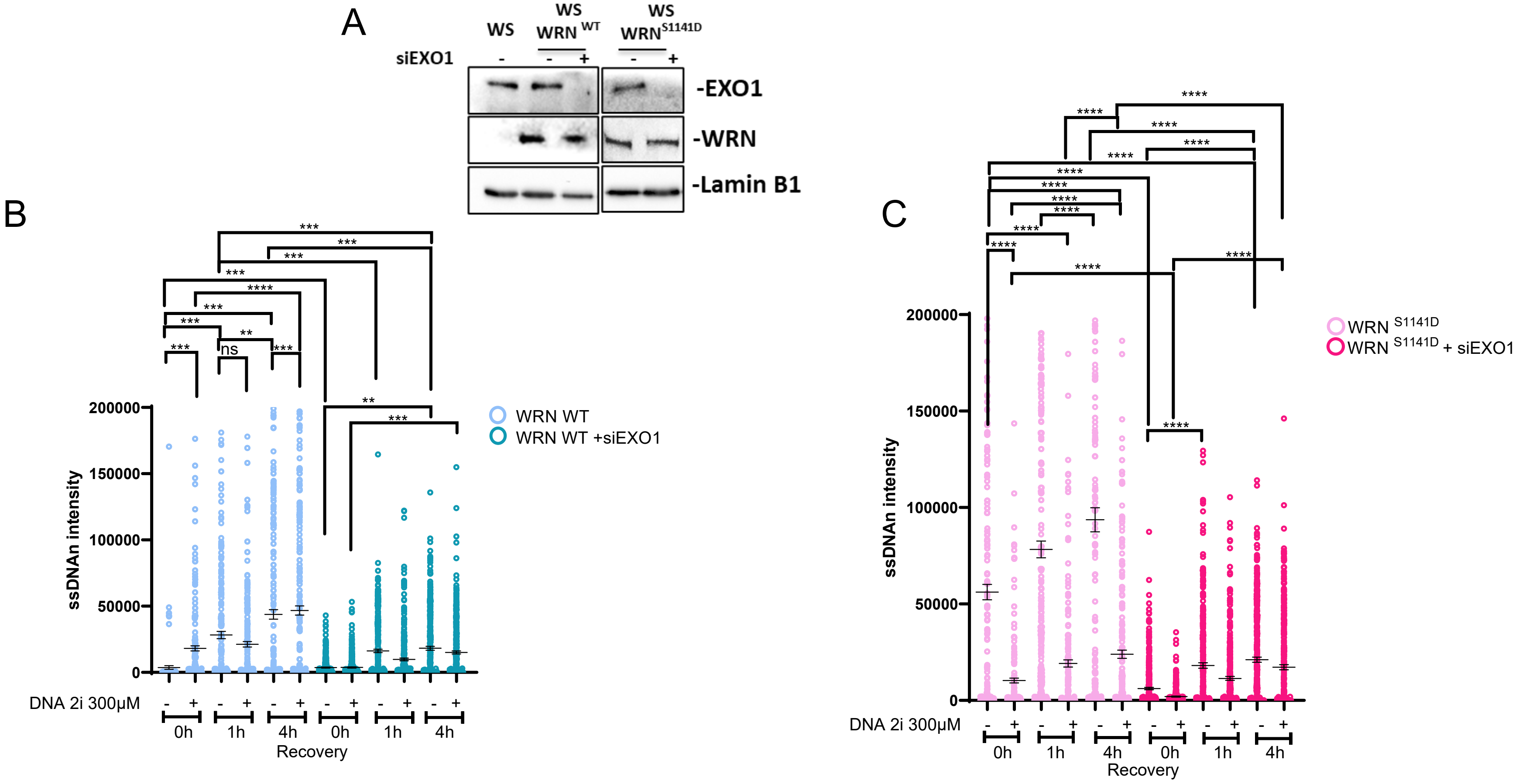

Supplementary Figure S9. Effect of combined S1133A and S1141A mutations on the formation of ssDNA during processing of CPT-induced DSBs

A) WB showing EXO1 depletion in WS-derived SV40-trasformed fibroblasts transfected with the wild-type or S1141D mutant form of WRN. B-C) WS-derived SV40-trasformed fibroblasts expressing the wild-type form of WRN or the S1141D mutant, transfected or not with siEXO1 oligos, were treated with 90min CPT in combination or not with DNA2i as indicated. The presence of ssDNA was analysed by non-denaturing IdU/ssDNA assay. The graph shows the mean intensity of IdU/ssDNA staining for single nuclei measured from two independent experiments (n=100, each biological replicate), bars show mean ± SE. Statistical analysis was performed by the ANOVA test (**** = P<0.0001; *** = p<0.001 ** = P<0.01; * = p<0.05; ns = not significant).

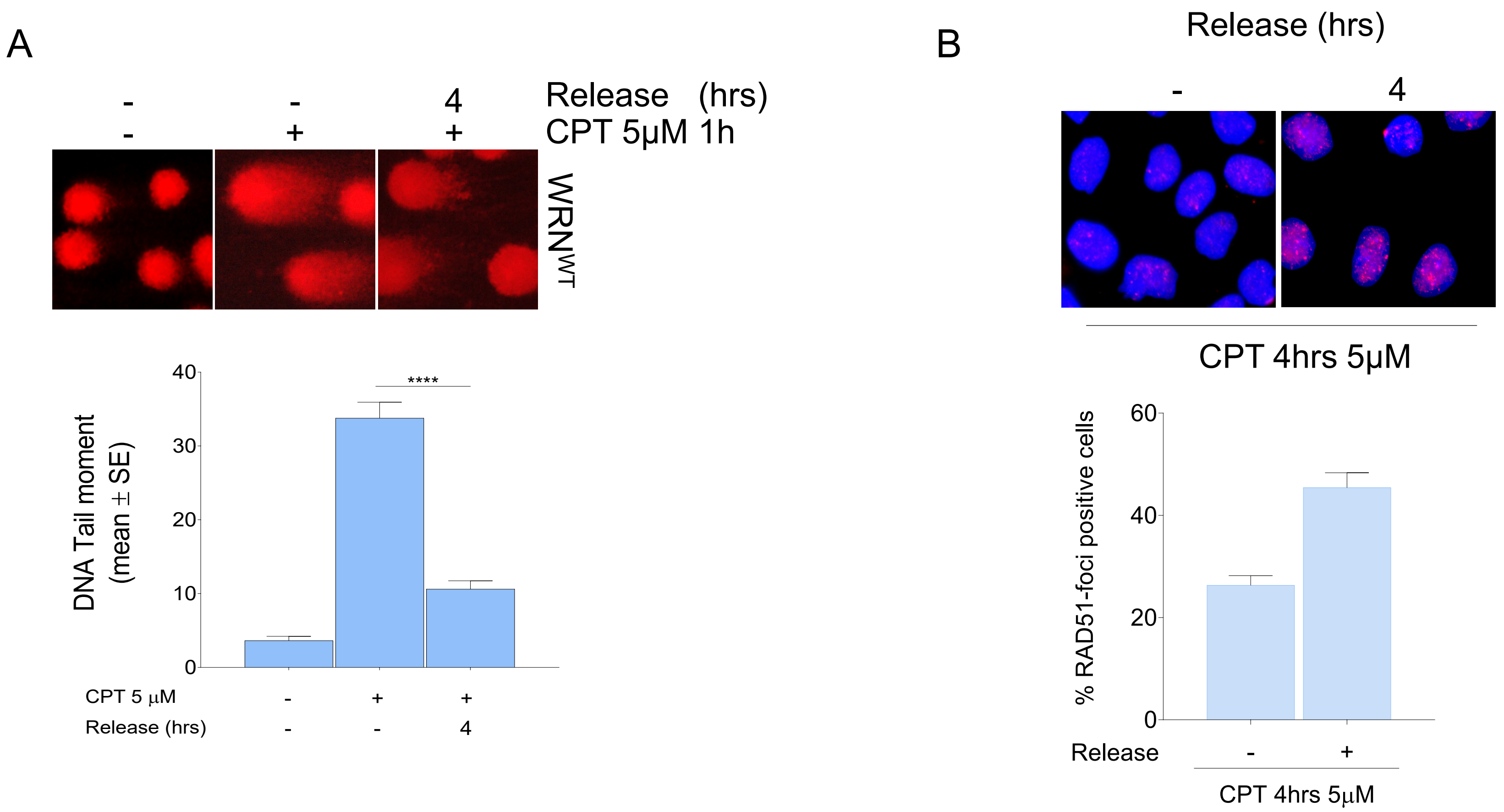

Supplementary Figure S10. Analysis of DNA repair and RAD51 recruitment during recovery from CPT

A) WS-derived SV40-trasformed fibroblasts stably expressing the wild-type form of WRN, were treated as indicated and DSBs evaluated by neutral Comet assay. The graph shows the percentage mean tail moment as obtained from two independent experiments (n=200, each biological replicate), data are presented as mean ± SE. Representative images are shown. Statistical analysis was performed by the ANOVA test (**** = P<0.0001).
B) Cells were treated as in “A” and analysed for RAD51-foci staining by IF. The graph shows the percentage of RAD51-foci positive cells as obtained from two independent experiments (n=200, each biological replicate), data are presented as mean ± SE. Representative images are shown.

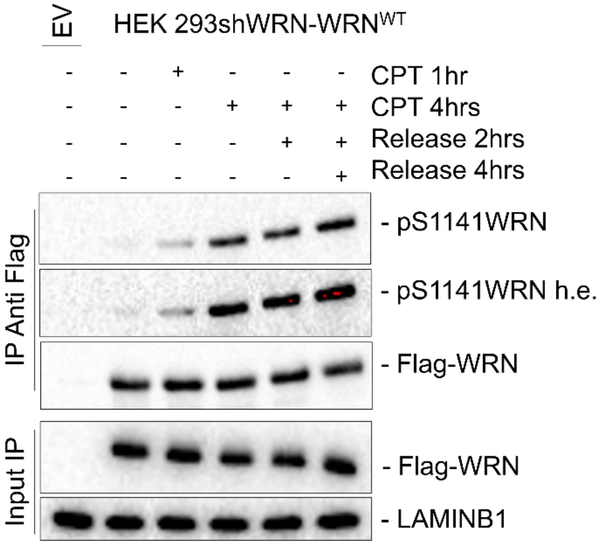

Supplementary Figure S11. Phosphorylation of WRN at Ser1141 is elevated during recovery from CPT-induced DNA damage

Cell were transfected with plasmid expressing the Flag-WRN protein and 48h after transfection, cultures were treated with CPT as indicated. Some cultures treated with 4h CPT were recovered as indicated. Cells were lysed and WRN protein was immunoprecipitated with anti-Flag-conjugated beads. Nine-tenth of IPs were analysed by WB with the anti-pS1141WRN antibody, while 1/10 was detected by anti-Flag antibody, as indicated. One-fiftieth of the lysate (input) was blotted with an anti-Flag antibody to verify transfection. An anti-Lamin B1 antibody was used as loading control. (h.e. = high exposure).

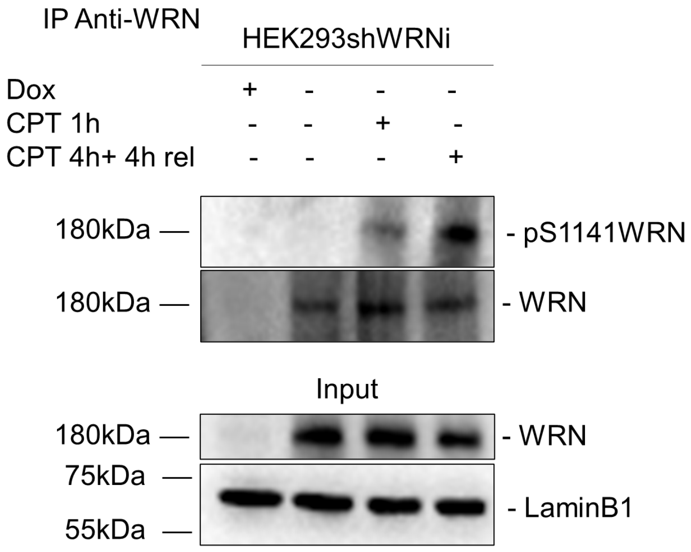

Supplementary Figure S12. Phosphorylation of WRN at Ser1141 is detectable also on endogenous protein during recovery from CPT-induced DNA damage

HEK293T stably expressing a Dox-inducible shWRN cassette were treated with CPT as indicated. Before being treated with CPT, some cultures were cultured in the presence of doxycycline to induce expression of WRN shRNA and silencing of the protein (This point represents the specificity control for the IP). Cells were lysed and WRN protein was immunoprecipitated with anti-WRN-conjugated beads. Nine-tenth of IPs were analysed by WB with the anti-pS1141WRN antibody, while 1/10 was detected by anti-Flag antibody, as indicated. One-fiftieth of the lysate (input) was blotted with an anti-Flag antibody to verify transfection. An anti-Lamin B1 antibody was used as loading control.

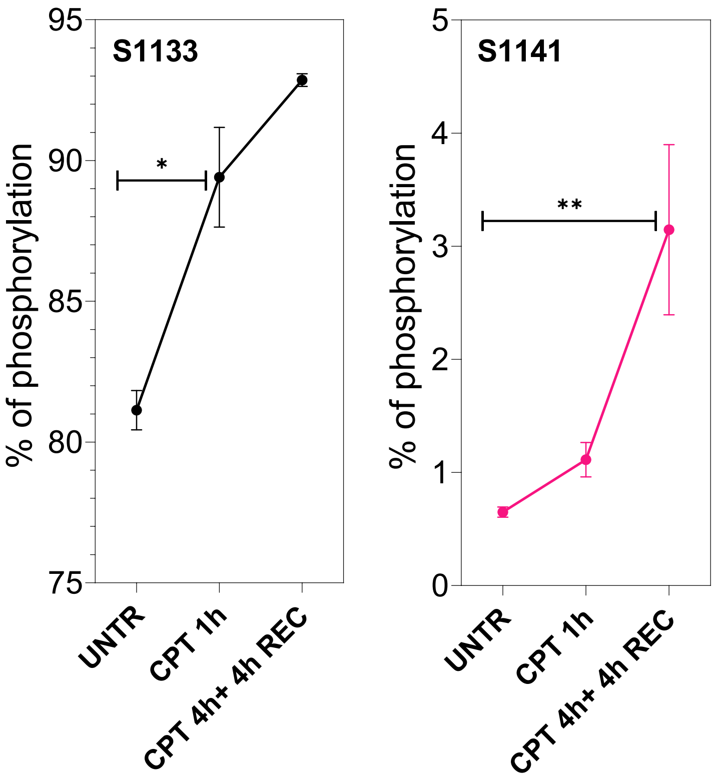

Supplementary Figure S13. Quantitative analysis of phosphorylation levels at Ser1131 and Ser1141 by Mass Spectrometry

Analysis of the percentage of peptides of WRN containing phosphorylation at the indicated residues normalized against the total number of peptides sequenced by Mass Spectrometry from three independent replicates. Where not indicated, values are not statistically different (Mann-Whitney test).

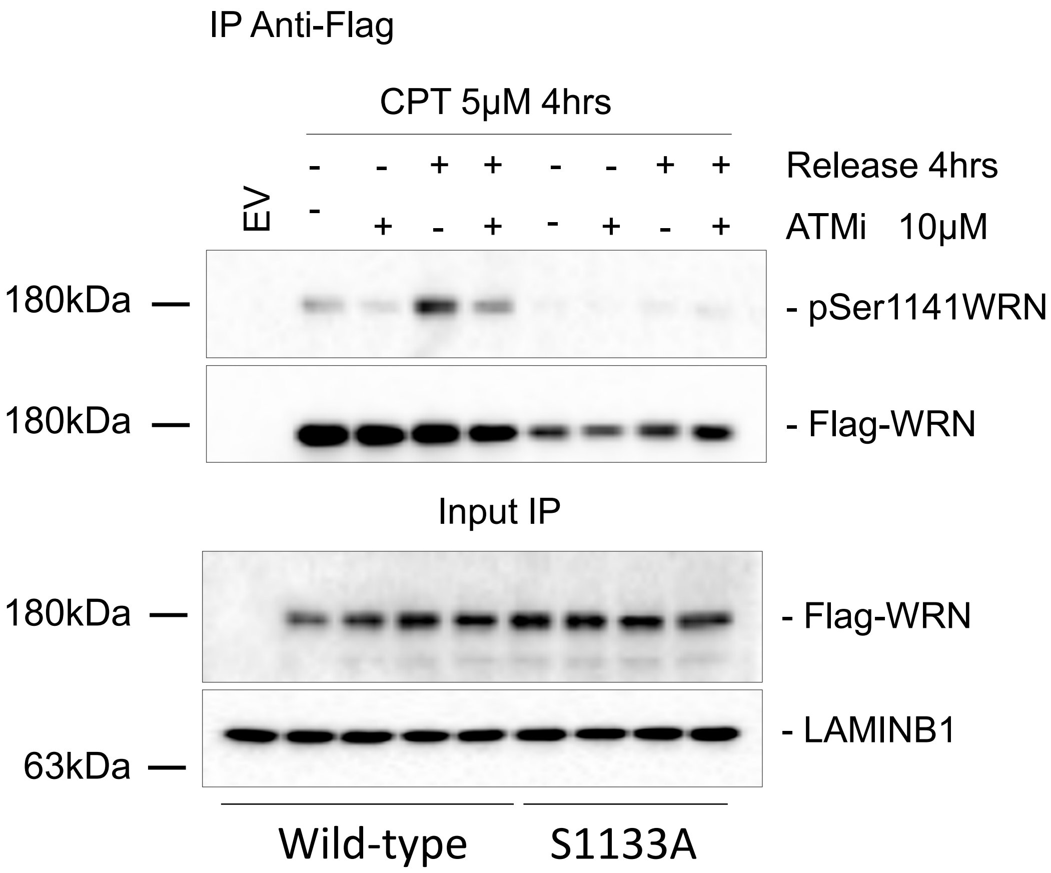

Supplementary Figure S14. Phosphorylation of WRN at S1141 requires prior phosphorylation at S1133

Cells were treated with ATMi (KU-55933) and CPT for 4 hours and then released for 4h. Cells were lysed and WRN protein was immunoprecipitated with anti-Flag-conjugated beads. Nine-tenth of IPs were analysed by WB with the anti-pS1141WRN antibody, while 1/10 was detected by anti-Flag antibody, as indicated. One-fiftieth of the lysate (input) was blotted with an anti-Flag antibody to verify transfection. An anti-LaminB1 antibody was used as loading control.

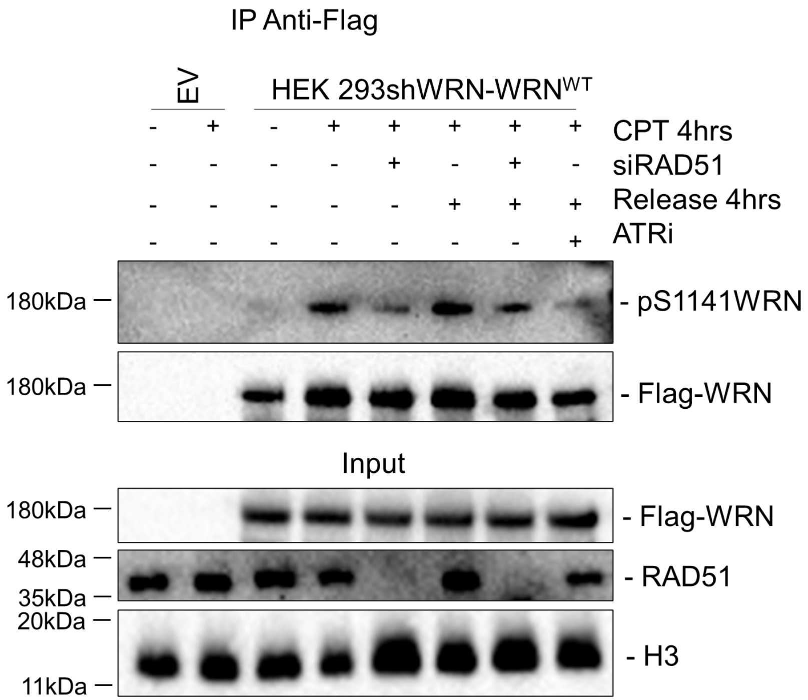

Supplementary Figure S15. Phosphorylation of WRN at Ser1141 during recovery from CPT-induced DNA damage requires RAD51

HEK293TshWRN cells were transfected with siCTRL or siRAD51 oligos and plasmid expressing the wild-type form of the Flag-WRN protein. After transfection, cultures were treated with CPT for 4 hours and recovered or not for additional 4hrs. Cells were lysed and WRN protein was immunoprecipitated with anti-Flag-conjugated beads. Nine-tenth of IPs were analysed by WB with the anti-pS1141WRN antibody, while 1/10 was detected by anti-Flag antibody, as indicated. One-fiftieth of the lysate (input) was blotted with an anti-Flag antibody to verify transfection and silencing. An anti-H3 antibody was used as loading control.

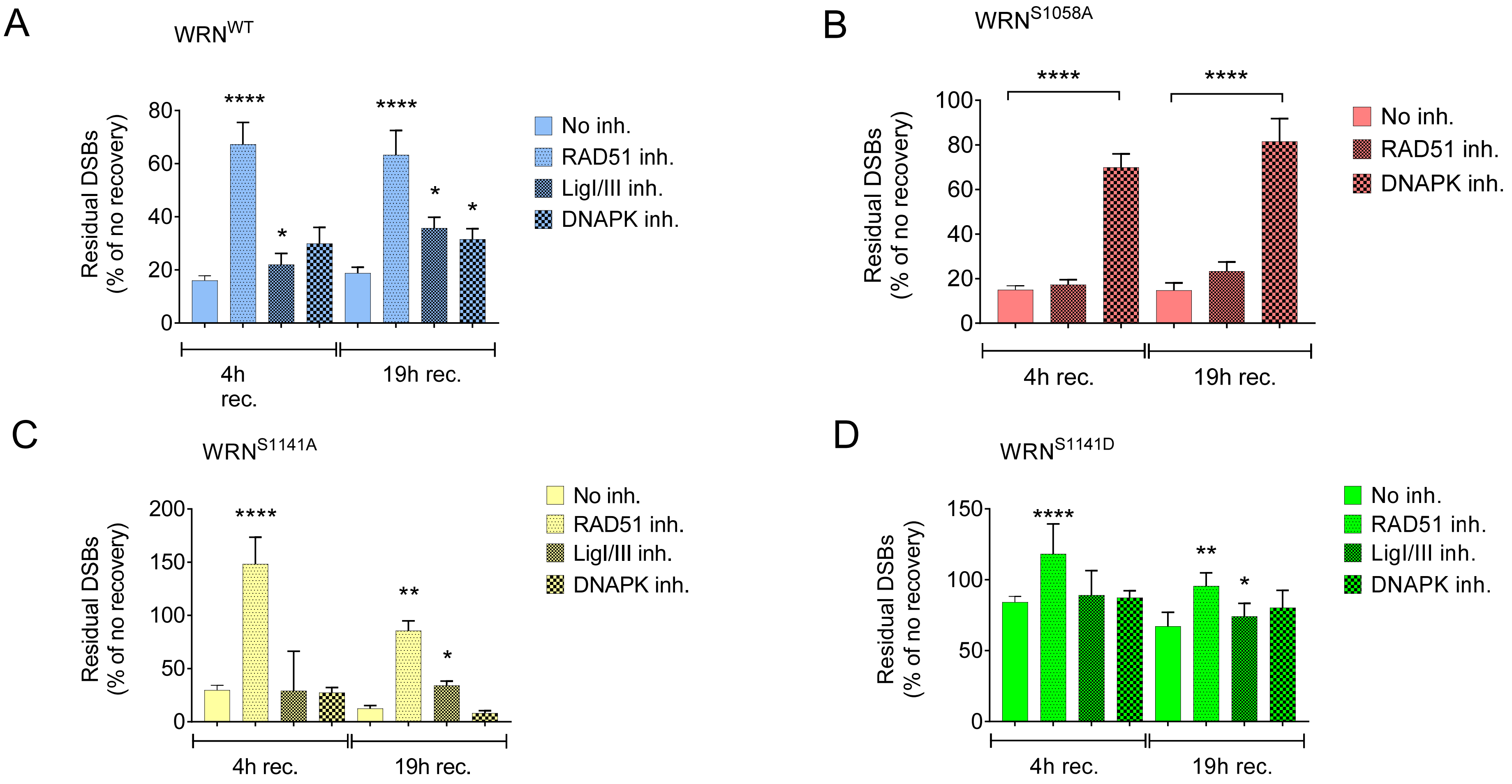

Supplementary Figure S16. Analysis of DNA repair during recovery from CPT

A) WS-derived SV40-trasformed fibroblasts stably expressing the wild-type form of WRN, the S1058A (B) or the two S1141 WRN mutants (C and D), were treated with CPT for 4 hours and DSBs evaluated by neutral Comet assay during recovery in the indicated DNA repair inhibitor, as indicated. The graph shows the percentage of the mean tail moment as obtained from two independent experiments (n=200, each biological replicate), ± SE. Statistical analysis was performed by the ANOVA test (**** = P<0.0001; *** = P<0.001; ** = P<0.01).

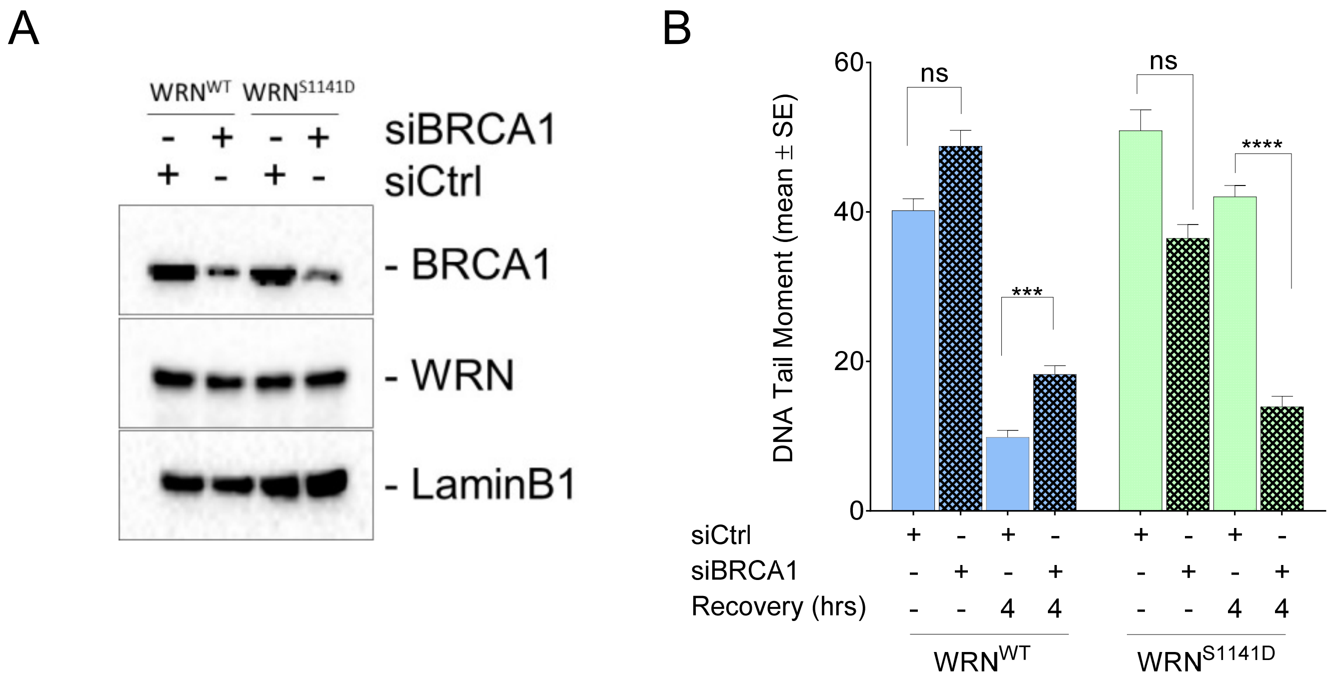

Supplementary Figure S17. Depletion of BRCA1 rescue DSBs repair in the S1141D-WRN mutant

A) WS-derived SV40-trasformed fibroblasts stably expressing the wild-type form of WRN or the S1141D WRN mutant were transfected with siCtrl or siBRCA1. WB shows the depletion efficiency. B) After transfection with siCtrl or siBRCA1 oligos, cells were treated 4h with CPT and recovered for additional 4h before being analysed for the repair of DSBs by neutral Comet assay. The graph shows the percentage mean tail moment as obtained from two independent experiments (n=200, each biological replicate), data are presented as mean ± SE. Statistical analysis was performed by the ANOVA test (**** = P<0.0001; *** = p< 0.001; ns = not significant).
